# How Reflective Automated e-Coaching Can Help Employees Improve Their Capacity for Resilience: Mixed Methods Study

**DOI:** 10.2196/34331

**Published:** 2023-03-10

**Authors:** Aniek Lentferink, Hilbrand Oldenhuis, Hugo Velthuijsen, Lisette van Gemert-Pijnen

**Affiliations:** 1 Department of Psychology, Health & Technology University of Twente Enschede Netherlands; 2 Marian van Os Centre for Entrepreneurship Hanze University of Applied Sciences Groningen Netherlands

**Keywords:** self-reflection, stress management, resilience, eHealth, self-tracking, e-Coaching, mobile phone

## Abstract

**Background:**

An eHealth tool that coaches employees through the process of reflection has the potential to support employees with moderate levels of stress to increase their capacity for resilience. Most eHealth tools that include self-tracking summarize the collected data for the users. However, users need to gain a deeper understanding of the data and decide upon the next step to take through self-reflection.

**Objective:**

In this study, we aimed to examine the perceived effectiveness of the guidance offered by an automated e-Coach during employees’ self-reflection process in gaining insights into their situation and on their perceived stress and resilience capacities and the usefulness of the design elements of the e-Coach during this process.

**Methods:**

Of the 28 participants, 14 (50%) completed the 6-week BringBalance program that allowed participants to perform reflection via four phases: identification, strategy generation, experimentation, and evaluation. Data collection consisted of log data, ecological momentary assessment (EMA) questionnaires for reflection provided by the e-Coach, in-depth interviews, and a pre- and posttest survey (including the Brief Resilience Scale and the Perceived Stress Scale). The posttest survey also asked about the utility of the elements of the e-Coach for reflection. A mixed methods approach was followed.

**Results:**

Pre- and posttest scores on perceived stress and resilience were not much different among completers (no statistical test performed). The automated e-Coach did enable users to gain an understanding of factors that influenced their stress levels and capacity for resilience (identification phase) and to learn the principles of useful strategies to improve their capacity for resilience (strategy generation phase). Design elements of the e-Coach reduced the reflection process into smaller steps to re-evaluate situations and helped them to observe a trend (identification phase). However, users experienced difficulties integrating the chosen strategies into their daily life (experimentation phase). Moreover, the identified events related to stress and resilience were too specific through the guidance offered by the e-Coach (identification phase), and the events did not recur, which consequently left users unable to sufficiently practice (strategy generation phase), experiment (experimentation phase), and evaluate (evaluation phase) the techniques during meaningful events.

**Conclusions:**

Participants were able to perform self-reflection under the guidance of the automated e-Coach, which often led toward gaining new insights. To improve the reflection process, more guidance should be offered by the e-Coach that would aid employees to identify events that recur in daily life. Future research could study the effects of the suggested improvements on the quality of reflection via an automated e-Coach.

## Introduction

### Background

Sustainable employability is, for a large part, negatively affected by stress, with one-third of work-related absenteeism among employees being caused by stress [1]. According to the European Union compass for action on mental health and well-being, more should be done in the preventative phase to increase employees’ capacity for resilience and reduce the risk of burnout [2]. Tackling stress at an early stage is vital because it can have negative consequences on health, well-being, and productivity [3]. To tackle stress at an early stage, it is necessary that employees cope effectively with the causes of the stress response (ie, stressors). Awareness about the stress response and the stressor is a prerequisite for employees to activate the desired behavior change, that is, effectively coping with the stressor. Moreover, employees also need to learn and select effective coping strategies to deal with the stressor [4]. Resilience is achieved when employees effectively deal with stress [5]. An employee’s capacity for resilience, defined as “the ability to bounce back after adversity” [6], is determined by the possession of several psychosocial and protective factors that influence the relationship between a stressor and the initial stress response. Examples of such factors are employees’ coping repertoires and emotion regulatory capacities [5].

Reflection is an important step in training employees’ capacity for resilience [5,7]. Reflection involves evaluating past experiences and learning from them with the aim of optimizing personal performance in future situations [8,9]. One of the ways in which reflection on stressful events improves resilience capacities is to prompt employees to search for ways to improve and adapt, recruit more coping strategies, and activate available resources such as social support or taking more time to complete a task [5]. It is useful to perform reflection soon after experiencing a situation that causes stress (reflection-in-action) and later (reflection-on-action) [10,11]. Stressful moments are opportune moments to perform a coping strategy, and a reassessment later in time can result in better recognition of stress or a stressor in future situations [5]. Another way in which reflection improves the capacity for resilience is that the negative event can be interpreted as less negative once time has passed, and individuals know the outcome of the stressor, which is often less severe than expected. This can lead to the situation being reframed into something more positive and unnecessary to worry about [12].

In traditional coaching settings, reflective coaching has received a great deal of attention as an effective and essential method to help coaches better understand and learn how to improve their situation [13,14]. The reflective coaching model [15], which is currently used in face-to-face coaching, includes four phases: (1) identification, (2) strategy generation, (3) experimentation, and (4) evaluation. *The identification phase* involves identifying issues worth solving and understanding why each of them is an issue; *the strategy generation phase* involves searching for and choosing possible solutions for the issue; *the experimentation phase* involves experimenting with the chosen strategies; and *the evaluation phase* involves evaluating the effectiveness of the strategy as a solution for the issue [15]. In short, reflection includes gaining awareness about the current situation and learning how to deal effectively with it or similar situations in the future.

Owing to the number of employees experiencing stress, labor-intensive face-to-face reflective coaching sessions to improve the capacity for resilience are not realistic [16]. eHealth technologies have the potential to coach users through the process of reflection without human involvement [17]. Self-tracking of stressful events and events related to resilience can result in awareness of the current situation [18]. Real-time measures of stress and resilience capacities (eg, heart rate variability) can be collected using self-tracking devices, such as smartwatches [19,20], or ecological momentary assessment (EMA) via smartphones. EMA “assesses individuals’ current experiences, behaviours, and moods as they occur in real-time and in their natural environment” [21].

eHealth tools that include self-tracking often present collected data in a graph for the user or as a simple summary. These persuasive technology features [22] can support users in observing their status and progress toward the desired behavior change [17]. However, previous research on self-tracking of health behavior indicates that awareness of one’s healthy lifestyle via self-tracking alone is not sufficient to effectuate the desired behavior change [18,23-25]. Through self-tracking alone, a great deal of the reflection process must be performed by the users themselves, such as gaining a deeper understanding of their current situation and deciding which coping strategy to apply. Cheo et al [23] stated that “the ultimate goal is to reflect upon one’s data, extract meaningful insights, and make positive change, which are the hardest part”. As described above, coaching during the reflection process performed by the user themselves is an effective and essential method to help employees extract meaningful insights and make positive changes [13,14]. End users and other stakeholders emphasized that coaching during reflection, in addition to the collection and summarization of data, was an important need for resilience training via eHealth technology [26].

Reflective automated e-Coaching has the potential to provide the necessary guidance that will aid in transforming awareness into behavior change. In this study, automated reflective e-Coaching is defined as supporting, advising, and guiding the user to evaluate past experiences and learn from these experiences for future improvement without the involvement of a human coach [9,27]. An automated e-Coach can personalize the coaching strategy based on self-tracking data and inputs from the user regarding their coaching needs, make use of persuasive features to motivate and stimulate behavior change [22], and be accessible 24/7 for users.

As we believe that reflective automated e-Coaching can affect behavior change, we aimed to study how employees using an automated reflective e-Coach perceive its *effectiveness* and *usefulness*. It is not only important to know the outcome of the guidance offered by the automated e-Coach, that is, its effectiveness, but also to gain an understanding of how the use of the different design elements of the automated e-coach and the interplay between them contributes to the outcomes, that is, the usefulness of the design elements during reflection [28]. To the best of our knowledge, no previous study has evaluated this aspect. To date, few eHealth technologies combine self-tracking and e-coaching. These technologies offer personalized feedback and goal setting based on self-tracking data [29-31]. However, they do not offer support, advice, or guidance during reflection, which is what automated reflective e-coaching fully entails in our opinion. The results of the perceived effectiveness and usefulness of reflective automated e-coaching can lead to implications for future designs in the context of resilience training. To explore this, we developed a prototype of the BringBalance app, as described in the section below.

The research questions that we aim to answer are as follows:

1. According to employees, what is the *perceived effectiveness* of the guidance offered by the automated e-coach in the BringBalance app during their reflection on the self-tracking data and strategies to improve their capacities for resilience?
  To what extent did employees gain insights into their current situation and strategies to cope effectively with current and future situations through the automated e-coach?
  How did employees perceive their stress levels and capacity for resilience before and after using the automated e-Coach in the BringBalance app?
2. What is the *usefulness* of the design elements of the automated e-coach in the BringBalance app to guide reflection by employees on the self-tracking data and strategies to improve the capacity for resilience?
  - To what extent are the individual design elements of the automated e-coach in the BringBalance app and the interplay between these design elements, useful during the process of reflection by employees?
  - What stimulating and stagnating factors did employees experience during the use of the design elements of the automated e-coach in the BringBalance app during their reflection process?

### The BringBalance App

The goal of BringBalance is to coach users through the process of reflection to strengthen their capacity for resilience. The app leads the user through the four phases of reflection from the reflective coaching model [15]. Each phase includes a set of modules in which users receive information via written text or videos and are asked to answer questions from the automated e-coach. Tools such as visualizations with summaries support the users in their reflection process. The BringBalance app is a product of “De Maar Training & Advies” and is based on their face-to-face coaching program, *Working on Resilience* [32]. Results from a pilot study on this face-to-face coaching program indicated positive effects on stress reduction [33]. In addition to the coaching program Working on Resilience, results from earlier studies on self-tracking and e-coaching for resilience training were also used during the design of the BringBalance app [26,27,34]. Other sources for creating the design of the BringBalance app were provided by the literature on reflection [10,12,15,24,35-37], coaching techniques [38-43], and persuasive design elements that can support the reflection process, such as visualization and personalization [17,22,44,45].

**Figure 1:**
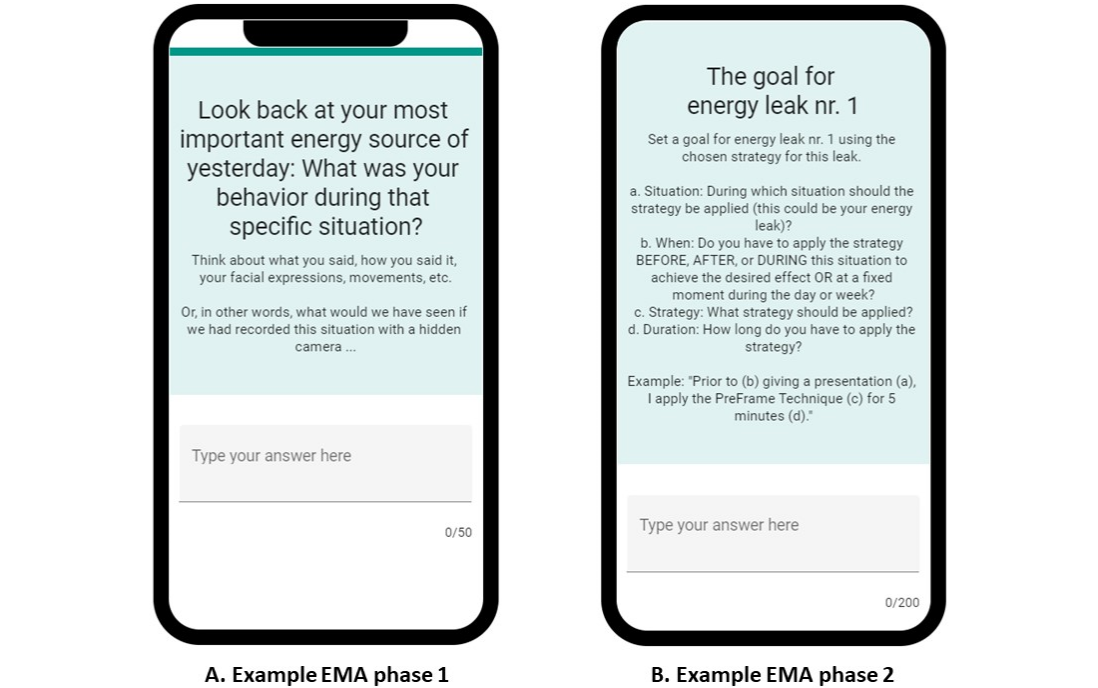
Screenshots of the BringBalance program phase 1 and 2 in The Incredible Intervention Machine (TIIM) app, including a few ecological momentary assessment (EMA) questionnaires.

**Figure 2:**
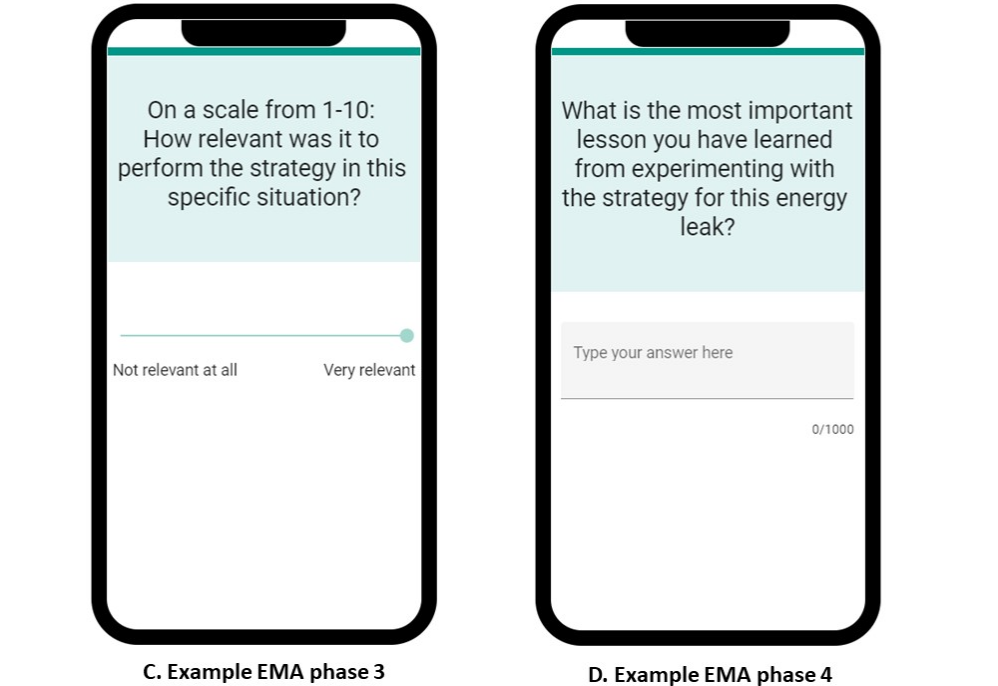
Screenshots of the BringBalance program phase 1 and 2 in The Incredible Intervention Machine (TIIM) app, including a few ecological momentary assessment (EMA) questionnaires.

The prototype of the BringBalance app was created using The Incredible Intervention Machine (TIIM), a tool of the Behavioural, Management and Social Sciences (BMS) lab at the University of Twente that supports building and testing eHealth interventions [46]. The BringBalance program via the app took 6 weeks to complete. The design elements were offered to the user in Dutch through the BringBalance program in the TIIM app, and all the design elements together comprised the automated e-coach. See Figures 1 and 2 for screenshots of the selection of design elements and Table 1 for an overview of the content of the BringBalance program. The design elements are in *italics* in Table 1.

The reflective coaching model with its four phases [15] was translated into a format suitable for automated e-coach. During the identification phase (phase 1), the employee was stimulated to gain insights into situations (energy leaks and sources) related to stress and resilience to find opportunities for improvement via several EMA questionnaires. The term energy leak was chosen to indicate bodily responses to stress that activate the sympathetic nervous system, such as a quickened heart rate and breathing pace, resulting in lower physical levels of energy [19]. In addition, in the context of this study, energy leaks refer to situations that lead to low mental energy levels, that is, a feeling of mental exhaustion. The term energy source indicates those resources that activate the parasympathetic nervous system, lowering the heart rate and breathing pace, and are related to a higher level of mental energy. Energy sources can help one regain balance in one’s energy levels [47], that is, enable a person “to bounce back after adversity”—which is also the definition of the capacity for resilience [6,38].

Phase 2, the strategy generation phase, consisted of learning the six BringBalance techniques via short clips and training for the techniques a day later. These BringBalance techniques are based on exercises from the HeartMath Institute [48] and entail being attentive to one’s heart area and using one’s imagination to breathe in and out through it [33]. In addition, a heart rate variability (HRV) sensor (Inner Balance Trainer, HeartMath Institute), placed on the participant’s earlobe, provided the participant with biofeedback during the training. HRV biofeedback has been found to support self-regulation capacities [49]. The HRV indices enabled the participants to see any immediate effect of the technique on their HRV levels, which they could then use to adjust their performance. At the end of phase 2, users decided upon helpful strategies for their three most important energy leaks and energy sources with the help of the e-coach. These strategies could be BringBalance techniques, an energy source, or a self-chosen strategy [40].

**Table 1:** Content of the BringBalance programa.

Phase	Duration	Requested from the user in this phase
Phase 1—identification	2 weeks	Three times during the week and once daily during the weekend: filling in the *EnergyBalance questionnaire* (see Multimedia Appendix 1). Once daily: *reflecting on the collected data of the day before including the 4G scheme* [38] asking the user to provide a more detailed description of the situation as well as their emotional state, physical state, cognitions, and behavior during the situation. The collected data from the day before were presented to the user via a table and graph. End of phase 1: choosing the 3 most important energy sources and leaks from *a list with an overview of the collected energy sources and leaks*. Result: self-tracking data on the energy balance for comparison with phase 3, list of energy sources and leaks, and top 3 most important sources and leaks.
Phase 2—strategy generation	2 weeks	Every Monday, Wednesday, and Friday: learning 1 of *the 6 BringBalance techniques*. The day after the introduction of the technique: receiving a reminder to practice the BringBalance technique with the inner balance trainer to obtain biofeedback during training the techniques. End of phase 2: Choosing strategies for their 3 most important energy sources and leaks. While selecting a strategy, users could receive guidance via *the strategy database* with an overview of all BringBalance techniques and tips for application in daily life or via *e-coach’s guiding questions; setting implementation intentions* [40] in which the strategies were linked to the energy sources and leaks and *reminders* with these implementation intentions on self-chosen moments for phase 3. Result: strategies were chosen for the top 3 energy sources and leaks, implementation intentions were set including the strategies for the energy sources and leaks, and reminders were set with the implementation intentions.
Phase 3—experimenting	2 weeks	Daily: receiving *reminders* with the implementation intentions set in phase 2; experimenting with the chosen strategies (optional: using the Inner Balance sensor) according to the implementation intentions; evaluating the strategy for its effect with a *strategy evaluation form* after experimenting with a strategy, including questions on the effect of the strategy on mood and energy levels, and stimulators and demotivators; filling in the *EnergyBalance questionnaire* once daily. Result: data on the evaluation of the strategies, self-tracking data on energy balance for comparison with phase 1.
Phase 4—evaluation	1 day	At the end of the program: receiving the data collected in phase 3 via visualizations in *tables* and *graphs*; *evaluating if the strategies* helped to prevent or resolve energy leaks and helped to make more use of energy sources; *evaluating if the energy balance* improved; advice on how to continue working on their energy balance after completion of the program. Result: final reflection on the strategies and energy balance and advice on how to continue working on their energy balance.

^a^The design elements are shown in italics.

In phase 3, the experimentation phase, users experimented with the chosen strategies and received reminders to do so at self-chosen moments [40]. After applying the strategies in real life, the users were asked to evaluate the strategy using an EMA questionnaire. In addition, the users were asked to report their energy balance every day. Energy balance was defined for participants as the balance between mental and physical energy-absorbing processes due to energy leaks and the processes that give them mental or physical energy from the energy sources [47].

All collected data from phase 3 were visualized in a graph and table and presented to the user in phase 4, the evaluation phase. In phase 4, the user evaluated whether the chosen strategies were the right strategies for their energy sources and leaks and whether their energy balance had improved. A more in-depth description of the BringBalance app, including the BringBalance techniques and persuasive design elements in BringBalance per phase of reflection, complying with the CONSORT (Consolidated Standards of Reporting Trials) guidelines for the reporting of eHealth interventions [50], can be found in Multimedia Appendix 1 [51,52].

## Methods

### Participants

Participants were recruited via email sent to all employees by the Human Resources (HR) department of a software company with approximately 350 employees in the east of the Netherlands. The HR department informed potential participants about the objectives of the study, the BringBalance app, data collection and management, and the amount of effort requested for employee participation. Employees willing to participate were asked to fill in a web-based questionnaire with the validated Dutch version of the Perceived Stress Scale (PSS) [53-55] and an informed consent form. The inclusion criterion for participation was a score above 14 on the PSS, indicating a higher-than-average perceived level of stress [56,57]. This inclusion criterion was based on earlier studies performed by the authors [26,58], which showed that employees with a certain level of stress tend to have a higher motivation to complete the intervention owing to a higher expected benefit compared with employees with lower stress levels. Finally, participants were required to own an Android (version 5.0 or higher) or iOS (version 10.0 or higher) smartphone.

A total of 45 participants filled in the questionnaire, with a response rate of 13%. Because 15 HRV sensors were available, 30 participants were invited to join either one of two sessions: November 2018 (n=15) or January 2019 (n=15). Participation in the study was voluntary.

### Study Design, Data Collection, and Analyses

**Figure 3:**
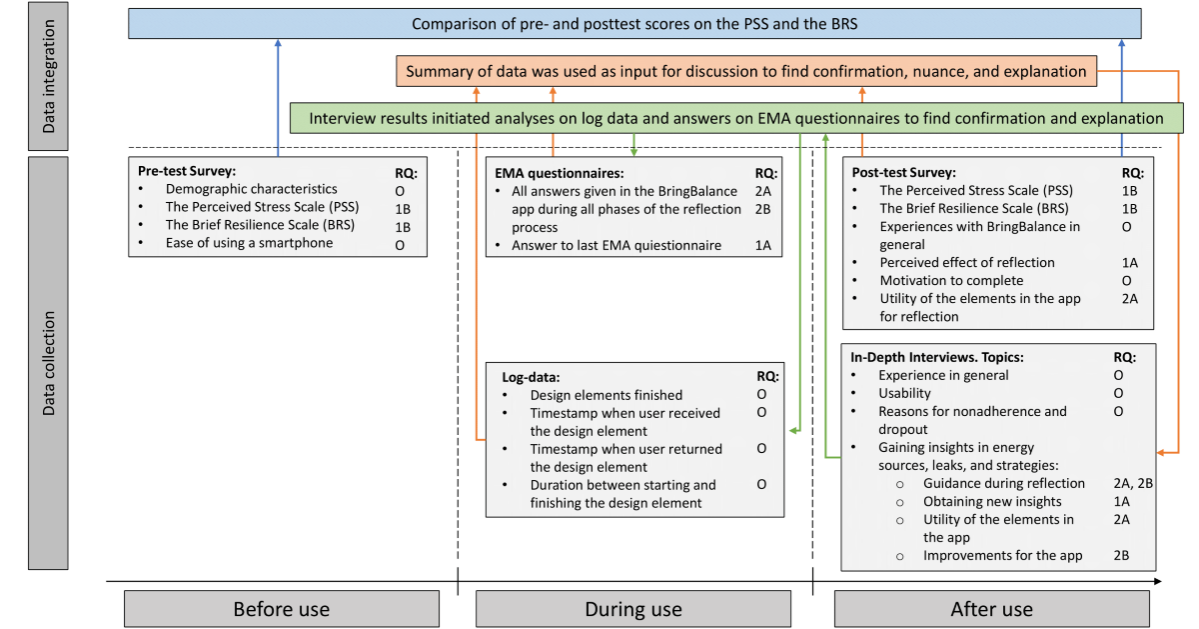
Flowchart of methods for data collection and data integration. BRS: Brief Resilience Scale; EMA: ecological momentary assessment; O: Other data to explain or nuance results; PSS: Perceived Stress Scale; RQ: research question.

A convergent mixed methods design was applied “to obtain different but complementary data on the same topic’ [59] for a more complete understanding of the problem [60]. The data collection included (1) a pre- and posttest survey, (2) EMA questionnaires in the BringBalance app, (3) log data, and (4) in-depth interviews. The pretest survey was completed before the BringBalance program; the EMA questionnaires and log data were collected during the BringBalance program; and the posttest survey and in-depth interviews were conducted after the BringBalance program. The collected data included perceived effectiveness (gaining insights [research question; RQ 1A], stress, and capacity for resilience [RQ 1B]) and perceived usefulness (utility of the design elements [RQ 2A] and stimulating and stagnating factors during the use of the design elements in the four phases of reflection [RQ 2 B]). The collected data included data on adherence to the intended use, dropout, app use, user motivation, usability, and experience with the BringBalance program in general. These data were used to confirm, explain, or nuance the results of the main outcomes of interest. Figure 3 shows a flowchart that includes an overview of the methods for data collection and integration of the data during collection and analysis. A data management plan was established according to the General Data Protection Regulation, a regulation for the protection of personal data inside and outside the European Union. More information on the data management plan can be found in the section *Data Management*.

#### The Pretest Survey

The web-based pretest survey was completed using Qualtrics survey software (Qualtrics, Provo, UT) 7 to 1 day before the start of the BringBalance program. The pretest survey included (1) demographic characteristics (age, gender, function, and educational level), (2) the Dutch version of the PSS; range of possible scores: 0-40) [53-55], (3) the Dutch version of the Brief Resilience Scale (BRS; range of possible scores: 1-5) [6,61], and (4) ease of using a smartphone rated on a scale from 1 to 5. The latter question was self-developed and included as an indication of the participant’s smartphone skills. Both the he PSS [53-55] and BRS [6,61,62] are validated questionnaires. PSS was used to check whether the participants met the inclusion criteria. The pretest PSS and BRS scores were used to gain insights into the study population and to compare against posttest scores to assess perceived effectiveness on stress and resilience capacities (see the blue box in Figure 3). However, no causal effect of the guidance offered by the automated e-coach could be deduced from the study setup. Data from the pretest survey were uploaded to the SPSS (IBM Corp) to calculate descriptive statistics.

#### EMA Questionnaires in the BringBalance App

During the BringBalance program, participants were asked by the automated e-coach to complete several tasks throughout the reflection process. Participants were asked via a reminder on their smartphones to fill in EMA questionnaires related to a specific task. The app included 17 different EMA questionnaires spread over the four different phases of reflection, each with their own content, doses, and timing. Some EMA questionnaires were released at fixed moments during the BringBalance program, whereas others were released based on a specific answer given in another EMA questionnaire. Multimedia Appendix 1 includes in-depth information on the setup of the EMA questionnaires, based on the reporting checklist from Van Berkel et al [63], along with examples of EMA questionnaires in the app. Figures 1 and 2 also include screenshots of the selection of available EMA questionnaires in the app. The answers to the EMA questionnaires provided insights into how users completed the reflection phases. These data provide insights into the perceived utility of design elements for reflection and stimulating and stagnating factors during reflection using the design elements. The last EMA questionnaire asked participants to report if they perceived a beneficial effect on their energy balance (yes or no) and if they had gained insights into their energy leaks and sources and strategies to improve their energy balance (yes or no). These data were used to determine whether participants gained insight into their current situation and strategies to improve their situation. Data were stored in the database of the BMS lab at the University of Twente and retrieved by uploading the data in Microsoft Excel files.

EMA data were used when it was necessary to further explore and interpret the results from the analyses of the interview data (see the green box in Figure 3). For example, when participants mentioned having difficulties interpreting a question from the automated e-coach, answers given on EMA questionnaires provided insights into the way users interpreted the question. In addition, EMA data were used as input for discussions during the interviews (see the orange box in Figure 3).

Answers to open-ended questions were gathered in Microsoft Word documents and uploaded into Atlas.ti (Scientific Software Development GmbH) for analyses using open, axial, and selective coding. Numeric scores were uploaded to SPSS via Excel files to calculate descriptive statistics.

#### Log Data

Log data were collected via the TIIM app during the BringBalance program and included the following data for each participant: (1) which design element was completed, (2) the timestamp when the design element was delivered to the user, (3) the timestamp when the design element was returned by the user, and (4) the duration of completing the design element. Log data were used to confirm, explain, or nuance the results of the main outcomes of interest (perceived effectiveness and usefulness). First, log data were used to analyze adherence to the intended use and dropout. The intended use was set up by one of the researchers (AL) and was based on the minimum expected necessary use to be able to go through the phases of reflection. See Table 2 for the intended uses. Insights into adherence to the intended use and dropout were necessary to gain an understanding if the perceived effectiveness (perceived effect on stress, resilience capacities, or gaining insight) may have been affected by factors other than the design elements of the automated e-coach, such as lack of ease of use, user motivation, or personal reasons for nonadherence or dropout. Elaboration of reasons for nonadherence and dropout during interviews helped to explain the perceived effectiveness of the automated e-coach and could reveal results on the perceived utility of design elements and stimulating and stagnating factors during the use of the design elements for reflection. Moreover, an overview of log data per participant was used during the interviews to discover the perceived utility and stimulating and stagnating factors during the use of different design elements (see the orange box in Figure 3). For example, when a participant never used an element*,* it could say something about the perceived utility of the particular design element during the four phases of reflection. In addition, log data were more deeply analyzed when the posttest survey and interview data at the group level identified a result that needed to be explored further (see the green box in Figure 3). The data were stored in the database of the BMS lab at the University of Twente and could be retrieved in Excel files. Excel files were uploaded to SPSS, and descriptive statistics were calculated, such as frequencies of adherence to the intended use per phase.

**Table 2:** Intended use for adherence.

Phase of BringBalance	Intended use
Phase 1—identification	The user completed 80%^a^ of the design elements “EnergyBalance” and “Reflection on the day before.” The user finished the design element “Top 3 most important energy leaks and sources.”
Phase 2—strategy generation	The user views 6 out of 7 (86%) short clips about strategies. The user chooses strategies for at least 2 energy leaks and 2 energy sources. The user sets implementation intentions for at least 2 energy leaks and 2 energy sources.
Phase 3—experimentation	The user completes 80%^a^ of the EnergyBalance questionnaires. The user completed at least 2 “strategy evaluation forms” per strategy.
Phase 4—evaluation	The user evaluates 2 strategies for energy leaks and 2 strategies for sources. The user evaluates the energy balance.

^a^No absolute values can be provided because the number of received design elements varied between users.

#### Posttest Survey

The participants were asked to fill in the web-based posttest survey via Qualtrics (Qualtrics) after they finished the BringBalance program and before they participated in the interviews. A total of 3 participants did not follow up on this because of time constraints. The full survey can be found in the Multimedia Appendix 2. The set of questions in the posttest web-based survey explored the following issues and was based on a survey used in an earlier study on the utility of persuasive design elements in an app for reflection [17]:

- The Perceived Stress Scale
- The Brief Resilience Scale
- Experience with BringBalance in general
- Motivation to complete BringBalance
- Perceived effect of the guidance offered by the automated e-coach on reflection outcomes: gaining insights into their energy balance and strategies to improve their energy balance
- The utility of the elements in the BringBalance app during the four phases of reflection

The participants reported their experience of using BringBalance in general by rating the BringBalance app on several aspects (scale of 1-10), such as usability, appeal, and integration into their daily life [64], as well as responding to three questions asking them to elaborate on their given ratings. In addition, the survey included two statements on their motivation to complete the BringBalance program. An example of a statement was *“The BringBalance programme motivated me to reflect on my energy leaks and sources*.*”* Insights into participants’ experiences with the technology and their motivation to use the technology were used to explain the underlying reasons for the perceived effectiveness and usefulness of reflection design elements [28,44].

Perceived effectiveness of the automated e-coach on reflection outcomes was measured in the posttest survey by three statements (5-point Likert scale from strongly disagree to strongly agree): (1) “The e-Coach has given me a clear overview of my most important energy leaks and energy sources”; (2) “Thanks to the e-Coach, I know what I could do in the future to prevent or resolve energy leaks”; and (3) “Thanks to the e-Coach, I know what I could do in the future to take more advantage of my energy sources.”

The main part of the survey consisted of questions regarding the experienced utility of the reflection design elements of the automated e-coach in the BringBalance app. Participants were asked to score the utility of each design element of the automated e-coach per phase of reflection that they received during the BringBalance program on a scale of 1 to 5. For example, “On a scale from 1–5, to what extent has the EnergyBalance questionnaire helped you gain insights into your energy leaks and energy sources? (1=not at all, 5=very much).” Each set of questions related to one phase of the BringBalance program ended with a blank space for participants to comment freely on the design elements of the automated e-coach for that specific phase.

The results of the posttest survey were used as inputs during the interviews (see the orange box in Figure 3). For example, the interviewee was asked to elaborate on the low scores given to the design elements of the automated e-coach. Posttest survey data were uploaded to SPSS, and descriptive statistics were reported for the group in total, which included the completers and noncompleters of the BringBalance program. Statistical analysis was not performed because of the small sample size (n=28).

#### In-Depth Interviews

Interviews were conducted one on one by the first author of this manuscript in person or via Skype (Microsoft) after the participants completed the BringBalance program. Recordings of the interviews lasted from 23 to 48 minutes. Furthermore, 7 of the 28 participants did not participate in the interviews because of practicalities.

In-depth interviews were held for confirmation and explanation, and to find nuances behind the answers given in the EMA questionnaires, the collected log data, and answers on the posttest survey (see the orange box in Figure 3). In addition, interviews were conducted to gain an understanding of the experiences, usability of the BringBalance app, perceived effectiveness of the automated e-coach, and how the process of reflection via the automated e-coach proceeded. The interview scheme was set up by the first author of this manuscript and finalized by all authors. The topics in the interview scheme were the user’s experiences in general, the usability of the app, reasons for nonadherence to the intended use and dropping out, the process of gaining insights into energy sources and leaks, related to the identification phase of the reflection process, and the process of gaining insights into when and what strategies to use, related to the strategy generation, experimentation, and evaluation phase of the reflection process. Subtopics for the reflection process included the design elements of the automated e-coach. The first 3 topics were discussed to obtain a sense of the experience with the app because experiences can affect the desired outcomes [28,65]. The elaboration of these topics by participants may also reveal the perceived utility of the design elements and stimulating and stagnating factors per phase of reflection. The latter 2 topics were discussed concerning the perceived effectiveness of reflection outcomes (ie, users’ insights into energy leaks and sources and strategies to improve their situation), the utility of the design elements of the automated e-coach and stimulating and stagnating factors during the use of the design elements. Results from EMA questionnaires, the posttest survey, and log data were used as inputs during the interviews (see the orange box in Figure 3). Participants were strongly encouraged to provide examples. The interview scheme can be found in Multimedia Appendix 3.

Interviews were transcribed verbatim. The transcripts were uploaded to Atlas.ti for qualitative data analysis. The coding scheme was created using inductive and deductive coding. Deductive codes came from the literature on reflection [15,35] and persuasive design elements [22] and included the design elements in the BringBalance app. Deductive codes for gaining insights from the participants were based on the level of reflection described by Durall et al [35]: no new insights, no reflection, recognition, and reflection. *No new insights* refer to insights that are a confirmation of what is already known, and *recognition* refers to quotes in which the user understands the data but acknowledges only what is expressed in the visualization of the data. *Reflection* involves gaining new insights via behaviors clearly associated with reflection, being surprised by the new insights, linking the insights to other experiences or situations in their daily life, or the insights affecting the beliefs or behavior of the user. *No reflection refers* to not obtaining any insights [35]. Open coding was performed for quotes that could not be labeled by deductive coding. Axial coding led to organizing codes into categories, removing synonyms, and splitting codes when necessary [66]. The initial coding scheme that resulted from coding the two transcripts was tested for intercoder consistency [67]. Two researchers (a student assistant, mentioned in the *Acknowledgments* section, and the first author of this manuscript) coded the two transcripts independently and discussed the differences until a consensus was reached. The discussions resulted in sharper descriptions of the codes. Finally, selective coding was performed to identify the themes that answered the research questions. During the process of selective coding, special attention was paid to finding contradictory quotes and differences between groups of participants, for example, between the study’s noncompleters and completers [66].

#### Mixing Strategies

Mixing strategies refer to those used to mix qualitative and quantitative strands [60]. All types of data collected were analyzed separately. As described above, some results from the analyses of one data source were inputted during the collection of another data source (eg, an overview of the log data per participant was used during interviews). Moreover, the results from different data sources per outcome of interest were compared to identify discrepancies and similarities between the results [60]. For example, the results on the utility of the design elements during reflection came from EMA questionnaires, posttest surveys, and interviews. This approach led to stronger evidence when similarities were observed and implications for further research when discrepancies were observed. Moreover, results from the analyses of one data source are often used to explain or nuance the results found during analyses of another data source.

### Data Management

The data management plan was made in DMPonline (TU Delft, Delft) and in collaboration with experts on data management from the Department of BMS, University of Twente, to ensure that data collection and storage were performed according to the General Data Protection Regulation. Ethical approval was obtained from the Ethical Committee of the University of Twente (reference number: P-1531727676).

## Results

### Demographic Characteristics

A total of 28 participants started using the BringBalance program, of which 21 (75%) were men and 7 (25%) were women, with a total average age of 36.5 (SD 9.7) years. Average PSS scores were 16.8 (SD 5.0) and BRS scores were 2.9 (SD 0.8). Table 3 provides an overview of the participants’ demographic characteristics.

**Table 3:** Demographic characteristics of the participants and their ease of using a smartphone.

			Study noncompleters (n=14)		Completers (n=14)		Total (n=28)
**Gender, n (%)**							
	Man	8 (57)		13 (92)		21 (75)	
	Woman	6 (43)		1 (7)		7 (25)	
	Nonbinary	0 (0)		0 (0)		0 (0)	
Age (years), mean (SD)			37.4 (11.2)		35.6 (8.3)		36.5 (9.7)
**Educational level, n (%)**							
	University of applied sciences	10 (71)		8 (57)		18 (64)	
	University	4 (29)		6 (43)		10 (36)	
Perceived Stress Scale score, mean (SD)^a^			16.4 (4.9)		17.1 (5.2)		16.8 (5.0)
Brief Resilience Scale score, mean (SD)^b^			3.2 (0.8)		2.7 (0.7)		2.9 (0.8)
Ease of using a smartphone, mean (SD)^b^			4.6 (0.5)		4.6 (0.5)		4.6 (0.5)

^a^Range of possible scores is 0 to 40.

^b^Range of possible scores is 1 to 5.

### Characteristics of Participants Not Taking Part in Interviews

Of the 28 participants, 7 (25%) did not participate in the interviews because of practicalities. Of these, 5 participants dropped out, of which 1 participant adhered to the intended use until dropping out. Other dropouts did not adhere to the intended use during all phases. The remaining 2 participants completed the BringBalance program and adhered to the intended use in phase 2. The average PSS score of the participants who did not participate in the interviews was 17.9 (SD 3.0), and the average BRS score was 3.0 (SD 0.6).

### Adherence and Dropout

**Figure 4:**
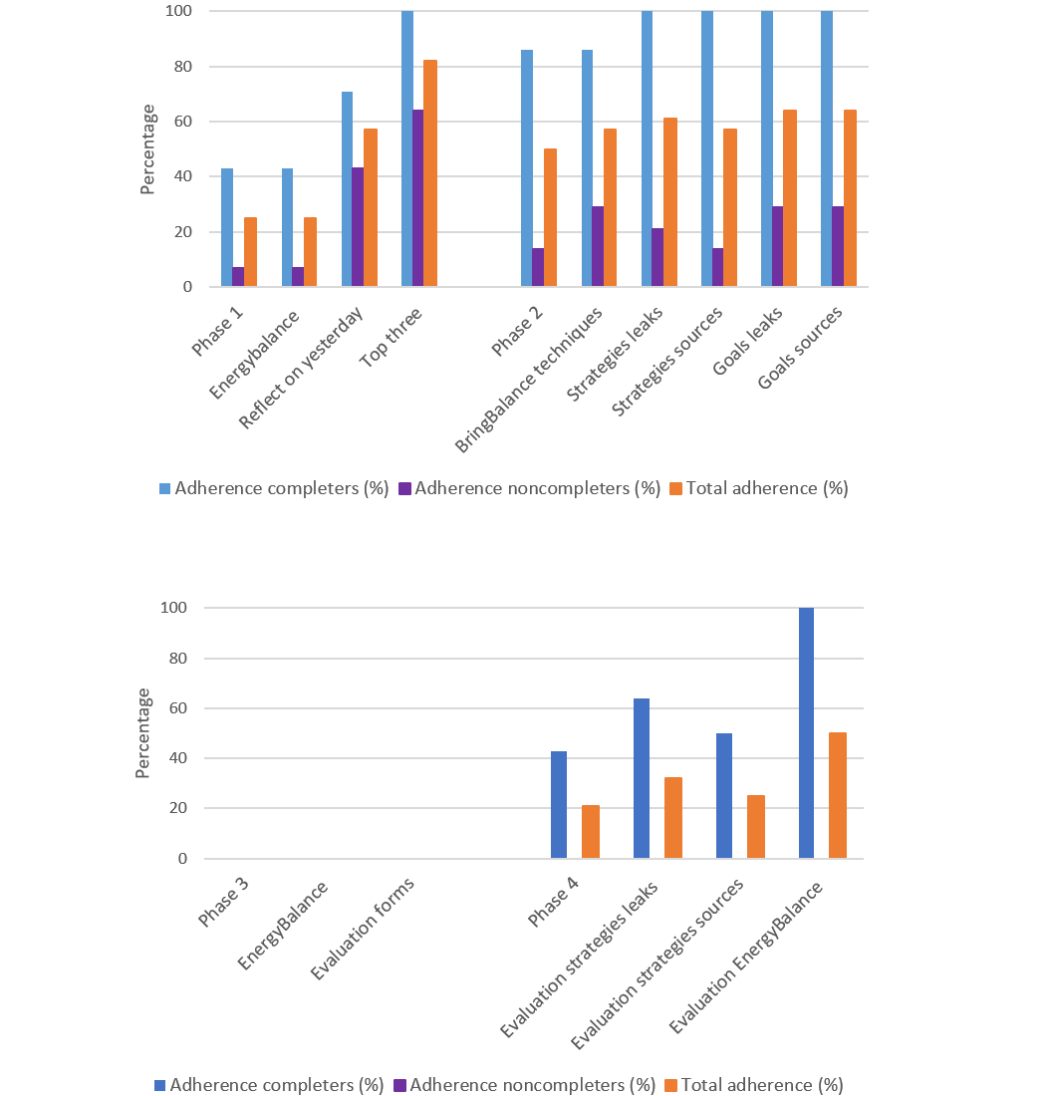
Adherence to intented use.

The log data indicated that none of the 28 participants adhered to the intended use, mainly due to an adherence rate of 0% in phase 3 (Figure 4). The adherence rates for the remaining phases were 25% (n=7) in phase 1, 50% (n=14) in phase 2, and 21% (n=6) in phase 4. The lowest adherence score in phase 3—experimentation—can be explained via interview data by a loss of overview by participants or their low-quality input in the earlier steps of the reflection process. According to the participants, the latter was a result of the guidance by the e-coach that steered them in a direction that was too specific (described in further detail in sections phase 1—identification and phase 2—strategy generation), lack of available time by participants, or the low priority given to the app. See Figure 4 for adherence rates among completers, study noncompleters, and the total group of participants.

A total of 14 participants completed the BringBalance program. Most participants dropped out in phase 2 (n=11, 39%). From the interview data and reports via email, the primary reason for dropping out was the program’s difficult integration into the daily life of participants owing to their full schedule (n=5, 18%), followed by the e-coach requiring too much of their attention and time (n=3, 11%), personal circumstances (n=3, 11%), or loss of interest in the program (n=3, 11%).

### Motivation to Complete and Ratings of the BringBalance Program

On average, participants rated the BringBalance program 6.5 out of a score of 10 (SD 1.0) in the posttest survey. On average, the BringBalance program scored 7.4 (SD 1.1) on being informative, 5.3 (SD 1.5) on usability, and 4.3 (SD 1.4) on integration in daily life (scale 1-10). Participants rated their motivation to reflect on energy sources and leaks as 3.3 (SD 1.0) and motivation to reflect on strategies as 2.7 (SD 0.7; scale 1-5). See Table 4 for an overview of participants’ ratings of the BringBalance program in general and their motivation to complete the BringBalance program, as determined by their scores in the posttest survey.

**Table 4:** Results of the posttest survey on participants’ ratings of the BringBalance program in general, their perceived effectiveness of the e-coach in the BringBalance program, and their motivation to complete the program.

Question^a^				Study noncompleters (n=14)		Completers (n=14)		Total (n=28)
**BringBalance app in general (scale 1-10), n (%)**								
	Score BringBalance in general, mean (SD)		6.2 (0.8)		6.9 (1.2)		6.5 (1.0)	
	The appeal of the content of the app, mean (SD)		6.8 (1.2)		6.9 (1.0)		6.9 (1.1)	
	Perceived utility of the app, mean (SD)		5.6 (1.5)		7 (1.0)		6.3 (1.4)	
	Usability of the app, mean (SD)		5.5 (1.3)		5.1 (1.6)		5.3 (1.5)	
	Integration in daily life, mean (SD)		3.6 (1.5)		4.9 (1.1)		4.3 (1.4)	
	Informative, mean (SD)		6.7 (1.4)		7.6 (0.8)		7.4 (1.1)^b^	
	**Advise the app to a colleague, n (%)**							
		Yes	6 (43)		10 (71)		16 (57)	
		No	8 (57)		4 (29)		12 (43)	
	BringBalance met my expectations, mean (SD)		5.4 (1.7)		7.1 (1.1)		6.3 (1.6)	
**Motivation: (1=strongly disagree, 3=neutral, 5=strongly agree), mean (SD)**								
	The BringBalance program motivated me to reflect on my energy leaks and sources.		2.9 (0.9)^c^		3.8 (1.0)		3.3 (1.0)	
	The BringBalance program motivated me to reflect on my chosen strategies for my energy leaks and sources.		2.4 (0.5)^c^		3.1 (0.7)		2.7 (0.7)	

^a^Abbreviations of the questions were used in the table.

^b^n=20.

^c^n=13.

### Perceived Effectiveness of Reflection

Among completers, pre- and postintervention scores on the PSS were mean 17.1 (SD 5.2) before and mean 16.9 (SD 3.5) afterward and the scores on the BRS were mean 2.7 (SD 0.7) before and mean 2.9 (SD 0.6) afterward. Of the 14 completers, 10 reported in the last EMA questionnaire that they had improved their energy balance. The remaining 4 completers reported that they gained insight into their energy balance. The interview data showed that participants gained insights into their energy sources and leaks (level of reflection: reflection). An example of a quote that shows reflection is the following: “My girlfriend is quite outgoing and she likes to constantly do things. I noticed that that costs me a lot of energy and I did too little to recharge by tucking away in my own little world for half an hour and then just enjoy the social things again” (respondent #6, study completer). Some participants wondered whether they were on the right track with their reflective process (level of reflection: Recognition). “Am I now thinking in the wrong direction or do I make it bigger than it actually is?” (respondent #1, study noncompleter). The interview data demonstrated that many participants gained insights into adaptive coping strategies and had an idea of when to use these techniques in daily life. However, the actual integration of the techniques in daily life was experienced as challenging by participants because of difficulties in learning the techniques (level of reflection: recognition). Elaboration on the difficulties encountered during this integration process are described in section *Phase 2—Strategy Generation*.

### The Usefulness of Design Elements During Reflection

#### Overview

Multimedia Appendix 4 includes the scores of each design element of the automated e-coach for its utility for reflection per phase of reflection. Utility scores (scale 1-5) are described below for elements that were discussed intensively by users during the interviews, indicating that these elements were evoked a lot among the users.

Multimedia Appendix 5 provides an overview of the identified themes, that is, the stimulators and stagnating factors according to the participants, per phase of reflection, and the specific design elements of the e-coach. The most important ones, that is, mentioned by many respondents or those with a great impact on reflection outcomes, per phase of reflection are described below. The design elements of the automated e-coach are placed in *italics* in the text below.

#### Phase 1—Identification

According to the participants, the first phase of the BringBalance app was easy to complete independently. Most participants mentioned that they were able to gain an understanding of their energy balance during their reflection, as guided by the e-coach. Based on the interview data, an understanding of their energy balance was mostly obtained via *the list with collected sources and leaks* at the end of phase 1, in which the most common sources and leaks were often perceived as the most important sources of their energy balance. This design element received a mean utility score of 3.7 (SD 0.8). Contextual information about the situation related to the energy sources or leaks was necessary to reconstruct the situation from the previous day, especially when the user’s data showed little variance. *The table* with a visualization of the data collected from the previous day received a mean score of 3.6 (SD 0.8) and *the graph* a 2.9 (SD 1.1) in the posttest questionnaire.

During the interviews, 5 participants mentioned that *the 4G scheme questions* were superfluous. Another group of participants found the element useful. The 4G scheme included questions asking the user to provide a more detailed description of the situation, as well as their emotional state, physical state, cognition, and behavior during the situation. The average utility score of the 4G scheme was 3.3 (SD 1.0). Participants who found it useful described that reflection later in time led to the observation of more relevant aspects than reflection in close occurrence to the situation. In addition, the participants experienced that the questions stimulated an in-depth reflection on the source or leak. Moreover, 4 participants had difficulties in recognizing indicators for energy sources and leaks and therefore with filling in *the 4G scheme questions*. Some participants experienced that filling in *the 4G scheme questions* made them understand their indicators for energy sources and leaks and enabled them to be better indicate an energy source or leak in future situations. “Over time, you become more and more aware that your body reacts in a certain way” (respondent #17, study noncompleter).

A total of 3 participants mentioned that the guidance offered by the e-coach led to the identification of sources and leaks that were too specific. “The tool only focuses on such a micro-moment, and it will not zoom out to a category or something” (respondent #21, completer). Some participants believed that they could have gained a higher level of reflection if they had reflected on their self-tracking data in dialogue with another person.

#### Phase 2—Strategy Generation

According to the completers, the design elements for *learning the BringBalance techniques,* including short clips, were perceived as helpful in the process of understanding when and what strategies to use. During the interviews, the users mentioned that they were able to learn the principles of the techniques.

Reflected in the interview data, *practicing the techniques* were perceived as a crucial part of understanding which techniques are useful for their situation. However, practicing the techniques in daily life was experienced as somewhat difficult without the presence of a relevant situation in which the technique might be useful. “Usually, the conditions were not right for the technique to work. I would call it ‘dry swimming.’ [...] Then you rush practicing the technique and you don’t really practice anymore” (respondent #16, completer). Being attentive to indicators of sources and leaks, identified through *the 4G scheme* in phase 1, was mentioned by a few as a prerequisite to understanding when to apply the techniques in daily life. To master these techniques, many perceived 2 weeks as too short a time.

Of the participants that used the Inner Balance Trainer, 65% (11/17) reported in the posttest survey that they found it useful to receive *HRV biofeedback* practicing strategies. It convinced participants regularly of the potential effect of the technique on physiological stress reactions in future stressful situations, which was reflected in the interview data. Some participants had difficulties interpreting the results, were uncertain when to perform the measurements with the sensor or saw no change in scores before and during practicing as the scores were indicated as good from the start.

Often, users mentioned that *connecting strategies to the most important leaks and sources* stimulated their mental processes on how to integrate the techniques into their daily lives. Most participants said that they were able to choose strategies using the tools in the app. Log data showed that 11 people chose *the strategy database,* with an overview of the BringBalance techniques and their tips for application in daily life, as a tool to help them decide on a strategy and gave this element a mean utility score of 3.6 (SD 0.8). In addition, 5 people chose *the e-coach’s guiding questions* and gave this element a mean score of 4.7 (SD 0.6). One participant remarked on this specific tool *help via the e-Coach*: “Those questions helped to think a bit more towards a certain direction. That made me think: ‘What was my energy leak about?’ And based on that, I started searching for a technique in that direction” (respondent #6, completer). A few participants expressed doubts if the strategies they had chosen were the right ones for their sources and leaks.

The participants mentioned that poor input from previous phases made it difficult to decide on strategies that were sometimes irrelevant. Three participants mentioned that the identified leaks and sources were no longer relevant and 6 participants mentioned that they did not master the techniques in this phase. In addition, participants missed discussing this step with someone else who might have helped them determine whether they had made the right choice or advised them about other possible options.

Most participants found that the element, *setting implementation intentions*, in which the strategies were linked to the energy sources and leaks*,* stimulated their intention and mental process to integrate the techniques into their daily lives, although some found that the element steered them too much toward goals that were too specific.

#### Phase 3—Experimentation

The interviews revealed that many participants experienced difficulties during the experimental phase. Although the steps were experienced as logical in theory, they mentioned that leaks and sources did not recur anymore during phase 3 and that its duration was too short to experiment. “It is very difficult to get there within a week and a half. [...] You ask yourself, did that technique help? And you don’t know for sure, and think: Maybe it was only a coincidence that the conversation went a little better” (respondent #24, study noncompleter). Log data showed that many started this phase later than planned, leaving little room for experimentation.

On average, *personally set reminders* along with the *set implementation intentions* scored 2.4 (SD 0.8) on utility. During the interviews, participants mentioned that reminders related to leaks and sources that occurred randomly over time did not trigger their application of a strategy, as the reminders were not “just-in-time.”

The evaluation of strategies began in phase 3 by evaluating every moment they performed a strategy with *the strategy evaluation form* (utility score on the posttest survey: mean 2.7, SD 0.8). Some participants experienced these forms as too repetitive and generic. “I can imagine that with the Zzleep or Flex technique, different questions come in handy” (respondent #6, completer)*.* Depending on the specific strategy or situation, some participants said that they did not find it necessary to complete *the strategy evaluation form* each time. For others, *the evaluation forms* were a trigger to start the evaluation process.

#### Phase 4—Evaluation

Half of the participants who went through the elements of evaluation acknowledged the utility of evaluating strategies as a conclusion of the BringBalance program. However, almost all participants mentioned that they had insufficient data collected in phase 3 to perform a comprehensive evaluation of the strategies and their energy balance. Participants would have filled in more strategy evaluation forms in phase 3 if they knew in advance that they would later receive the collected data of these strategy evaluations as *visualizations of the collected data in a table and graph from phase 3*.

## Discussion

### Principal Findings

To improve the capacity for resilience through self-reflection, this study’s main aim was to examine the *perceived* *effectiveness* of the guidance offered by the automated e-coach in the BringBalance app during the reflection process on stress and resilience capacities among employees. In addition, this study’s goal was to determine the *usefulness* of the design elements of the automated e-coach for reflection, and the stimulating and stagnating factors during the use of the design elements.

#### Perceived Effectiveness

Pre- and posttest scores on perceived stress and resilience capacities were not significantly different among the completers of the BringBalance program. It should be noted that no statistical tests were performed because of the small sample size. Most completers reported an improved energy balance and insights into their principal energy leaks and sources as well as effective strategies for improving their situation. The reflection outcome of “linking these insights to other experiences or situations” by integrating the techniques into their daily lives was often not achieved.

#### The Usefulness of Design Elements for Reflection

Participants were easily able to reflect on self-tracking data and decide their most important energy leaks and sources with the design elements of the e-coach. Participants experienced difficulty integrating strategies relevant to their energy leaks and sources into their daily lives and reflecting on whether their chosen strategies were the right ones with the design elements of the e-coach.

Important stimulators for the process of reflection on self-tracking data were the design elements of the automated e-coach that stimulated the re-evaluation of situations and the observation of trends in the collected data through the breakdown of the reflection process into smaller steps and visualizations, including visualizations of the data via *a table with an overview of sources and leaks from the previous day,* and *a list of sources and leaks at the end of phase 1*. Some participants experienced that the re-evaluation later in time led to the ability to gain a larger perspective, leading to their understanding of more relevant details of a situation. In addition, contextual information added to the visualizations about the situation related to the energy source or leaks was necessary to be able to re-evaluate the situation later in time.

A stagnating factor for some participants was that the guidance offered by the e-coach led to the identification of sources and leaks that were too specific. Although most participants found it easy, some had difficulties recognizing physiological, mental, and emotional indicators of sources and leaks. These indicators are required in *the 4G scheme.*

The important stimulators for the process of reflection on strategies were (1) *the short clips* in which the participants learned the principles of the techniques, (2) the heart rate variability *biofeedback* to help them understand the principles and stimulate the effect of the BringBalance techniques on physiological stress reactions, (3) design elements that stimulated *practicing the techniques* because this rehearsal was perceived as a crucial step in the reflection process, and (4) *the tools to help them decide upon the strategies* and *set up implementation intentions* as these elements stimulate the user’s mental process on how to integrate the strategies into their daily lives. Participants found it useful to link the strategies to the sources and leaks, although, in practice, this did not bring about the desired results.

The most important stagnating factor for this lack of success was the low-quality input from previous steps in the process, such as the very specific energy sources and leaks identified in phase 1. The design elements to *set up implementation intentions* and *reminders* tended to lead participants excessively toward a specific context in which the strategy should be performed. In practice, this left little room for experimentation as the situation often did not recur. In addition, many participants experienced a lack of mastering the techniques in their daily lives owing to perceived time constraints, lack of relevant situations in which to practice, and doubts about performing the techniques in the right manner. These factors led to little experimentation and data collection in phase 3 and, therefore, difficulties for evaluation in phase 4, which involved answering the question whether the strategies were the right ones for dealing with the participant’s energy sources and leaks.

### Comparison With the Literature

#### Perceived Effectiveness

In contrast to this study’s results indicating that scores on perceived stress and resilience were not much different in pre- and posttest scores, Rijken et al [33] observed a tendency toward improvement in stress-related outcomes for the face-to-face program on which BringBalance was partly based. It should be noted that no statistical analysis could be performed in the BringBalance study owing to the small sample size. In addition, the prototype of the BringBalance program used in this study scored rather low on usability and integration in daily life, which likely affected the effectiveness of the guidance offered by the automated e-coach in the BringBalance app during the reflection process [65,68]. Still, there is a possibility that the element of reflection via human dialogue has played a role in the differences observed in the effectiveness of stress measures between the results of Rijken et al [33] and this study, as this element was an important difference between the two programs. Some participants also mentioned the potentially stimulating role of human dialogue during reflections. The elaboration on how to deal with this issue in future designs is further discussed below.

#### The Usefulness of the Design Elements for Reflection

An important stimulating factor in the reflection process guided by e-coach seems to be the breakdown of the reflection process into smaller steps. These steps seemed to trigger participants to rethink their situations, which led to the observation of trends and a deeper understanding of their indicators of stress and resilience. The same process was observed in a study by Isaacs et al [12], who found that participants defined as recorders (those who reported the event once) and those defined as reflectors (those who reflected on the event multiple times), both benefitted from their reflections, although reflectors were more likely to observe patterns and learn from these events to improve future performance.

Three important stagnating factors during the reflection process were (1) difficulties participants had in recognizing indicators for the presence of energy sources and leaks; (2) the identification of specific energy sources, leaks, and implementation intentions as guided by the e-coach; and (3) a perceived lack of availability. Although these stagnating factors were not experienced by all participants, targeting them can significantly impact reflection outcomes in a positive way for participants who experienced them.

First, participants in this study elaborated on the positive effect of being consciously aware of physiological, mental, and emotional indicators for their sources and leaks, including (1) being better able to recognize the presence of a source or leak in the future and (2) to identify opportunities for applying a strategy, known as “trigger identification” in the Systematic Self-Reflection Model of Resilience. This model emphasizes the importance of self-reflection in the process toward resilience [5]. Moreover, reflection on cognitions and emotions can help explain the behavior of the participant in a situation of interest and can lead to a higher level of understanding of their situation [38]. However, some participants in this study were not consciously aware of their indicators during the situation, that is, reflection-*in*-action, or found it difficult to reproduce the physiological, mental, emotional, and behavioral indicators concerning the situation when it occurred the next day, that is, reflection-*on*-action. This difficulty can negatively impact their reflection outcomes [38].

To identify the indicators effectively, both reflection-in-action and reflection-on-action are important [10,11]. Difficulties with reflection-in-action can be the employee’s limited ability to reflect under high levels of stress [5] or the concept of alexithymia because not everyone can recognize emotional responses [69]. Alexithymia can also explain difficulties with reflection-on-action because attention increases the likelihood of recalling the situation later [70]. In addition, other factors that negatively affect recall can explain difficulties with reflection-on-action, such as motivation and fatigue [71]. Proper guidance during reflection-in-action can solve problems with reflection-on-action, and vice versa. For example, problems due to alexithymia or recall may be solved by notifying the user just in time about the presence of stress symptoms and stimulating them to pay conscious attention to triggers in action [72]. Moreover, as mentioned by the participants, contextual information is necessary to recall the situation a day later and making notes in close occurrence is one method that seems to effectively tackle recall problems [10]. In this study, reflection-on-action was perceived as useful by participants because it enabled them to observe more details later in time. Reflection-on-action can also positively affect one’s overall reflection as the initial intensive stress response is diminished [5].

Second, the automated e-coach in the BringBalance app stimulated the participants’ intention to do something about the situation. However, a loss of relevance to continue behavior change was experienced when the identified sources and leaks were too specific. The problem of the limited applicability of previously collected data on well-being to current situations has been observed more often [24]. One way to maximize the applicability of specific situations to current situations might be to start choosing a strategy based on the underlying values and personal goals of the identified sources and leaks [5]. Situations that involve a mismatch between the current coping strategy and personal values and goals increase the need to do something about the situation [5]. Therefore, the underlying goals and values may serve as trigger points for adaptive coping strategies. The increased chances of recurrence can also lead to more opportunities to practice the techniques, which was mentioned by participants as a crucial step and is acknowledged in the literature as well; “The strengthening of resilience is a process of experiential learning and more speciﬁcally learning through reﬂection on doing” [5].

Finally, it is unlikely that participants were constrained by the actual time needed to interface with the BringBalance program, which was approximately 15 minutes per day. This response was more likely a result of perceived time constraints caused by their busy schedules. This conclusion is based on the low scores given by the participants regarding the integration of the BringBalance program into their daily lives. Moreover, in the context of work, it seems that employees prioritize work-related activities over resilience [58] or learning via reflection [73].

### Strengths and Limitations

First, our study population consisted of participants with high educational levels. As reflection relies on the analytical skills of a participant [74], it might be that the performance during the reflection process and the need for guidance from the automated e-coach by our study participants are different for the overall working population.

Second, the sample size was too small to conduct statistical analysis of pre- and posttest scores on perceived stress and resilience capacities and differences in scores given by study noncompleters and completers on the utility of design elements. This limitation restricted the strength of some of our conclusions. Although the statistical power was low, this study’s results did meet the primary aim of this study, namely to explore the potential of guidance offered by an automated e-coach during the participant’s reflection process for resilience training and to ascertain implications for future designs based on the results; therefore, valuable insights that can support future design were obtained.

Third, although low adherence rates are common for prototype versions of eHealth technology, none of the participants precisely adhered to the intended use. On the one hand, low adherence distracted the user from the original goal of the program, which was to reflect on improving resilience capacities, and likely affected the effectiveness. On the other hand, reasons for low adherence revealed important stagnating factors for reflection guided by the automated e-coach, such as the loss of relevance to continue owing to specific energy leaks and sources. Moreover, it should be noted that the setup of adherence to the intended use was based on 1 researcher’s expectations of the minimum necessary use by the user. This expectation may have been too ambitious as no participant adhered to the intended use, and results indicated that most participants gained insights into their energy leaks, sources, and strategies to improve their energy balance.

Finally, 7 participants were not involved in the interviews owing to practicalities, which might have affected the validity of the qualitative results. A relatively higher number of study noncompleters were observed among noninterviewees, and the noninterviewees’ PSS scores tended to be somewhat higher in comparison with the interviewees, although statistical tests could now be performed. However, similar characteristics were observed among interviewees, as 9 were study noncompleters and 7 scored higher than the average PSS score of the noninterviewees. This suggests that the validity of the qualitative results was not significantly influenced to a large extent.

Regarding the strengths of this study, the first is that the BringBalance program’s design was strongly based on literature and was created in close collaboration with stakeholders. These 2 aspects increase the chances of improving uptake and creating an impact on eHealth technology [65]. The participants perceived the design decisions made for the content in the app as logical and interesting. The usability of the app and its integration into daily life are points of attention. This can be explained by the limited options in the way the prototype could be developed. Usability and integration issues can be overcome when an app is developed with a higher level of fidelity [68].

Second, a mixed methods approach was used in this study. Results from one data source were used during the collection of another data source (eg, log data were used as inputs during interviews) or results from one data source were a trigger to explore more profoundly into the data from another source (eg, to review the log data to explain the lower scores given on the guidance offered by the e-coach by study noncompleters in comparison with completers). This enabled us to confirm or question the results of one approach to another. In addition, it enabled a deeper interpretation of the results by finding nuances in the data from other approaches.

### Implications for Future Design and Research

To our knowledge, this study is the first to provide insights into the design elements of an automated e-coach that can simulate the self-reflection process, from the identification of relevant events to the evaluation of strategies [15], without support from a human coach. Future design and research can begin by focusing on the effects of making more and better use of persuasive features during the reflective automated e-coaching process, based on the 3 stagnating factors described above. Persuasive features can stimulate users’ motivation for behavior change and are shown in *italics* in the discussion below [22].

First, as described above, trigger identification is an important aspect of the reflection process and can result in both reflection-in-action and reflection-on-action. Continuous biofeedback, a form of persuasive *self-tracking* feature [22], creates a unique opportunity to receive timely external feedback [75] when stress is present. Moreover, biofeedback can be used to determine when the intensity of the stress response is diminished to some extent, which could have a positive effect on the quality of one’s reflection [5]. Several commercially available wearable devices are capable of continuous measurement of the physiological responses related to stress and resilience capacities, such as HRV measurements [19,76]. These measures can indicate within minutes that stress is present or when stress is decreased and, hence, signal to employees their capacity for resilience [76].

Second, the automated reflective e-coach should offer guidance in translating specific events into overarching goals and values that recur in daily life. The e-coach can help the user split the complex behavior into a higher perspective that oversees the collected data and breaks it into short and simple tasks, which is related to persuasive feature *reduction* [22]. For example, the e-coach can ask the user to answer additional questions regarding their underlying opinions, values, qualities, and drivers to learn and understand their goals and values in daily life [74]. This implication for future design can also improve the technology’s effectiveness on the desired behavior change and user motivation, as this way the content better suits the user’s context of working and living [22,26].

Third, we propose a more dynamic process in which users can decide the pace of completing a phase, related to the persuasive feature of *personalization*, thereby avoiding poor inputs from previous steps in the reflection process owing to perceived limitations in time.

Finally, some participants believed that reflection in a dialogue with another person would lead to higher levels of reflection. This dialogue was also desired by participants to eliminate personal doubts about the individual reflection process. The involvement of a professional coach limits the program’s scalability. Therefore, the first implication is that peer groups within organizations could facilitate dialogue. These peer groups can be organized according to the persuasive features of *social facilitation*. Using this feature, users can contact peers through the app [22]. Previous literature has found that peer guidance during reflective practices improves the reflective process [77,78]. Second, automated e-coaches could match human-to-human dialogue to a greater extent. This technological development is currently on the research agenda [79,80]. To match a human-to-human dialogue, the e-coach should have high *surface credibility* via fluent dialogue, and the user must experience the e-coach as a real human, an achievement that still requires considerable research and testing. However, some persuasive features that are rather easy to implement can improve the *surface credibility* of the currently available automated e-coaches by applying a high level of *personalization,* for example, by regularly selecting coaching messages based on previous inputs given by the user or repeating these inputs in messages, and the e-coach should adopt a *social role*, for example by greeting the user by name [22,79,80].

A follow-up study using an updated prototype of higher fidelity, including these aspects, can be performed to test the effects of the guidance offered by the automated e-coach on stress and resilience capacities, and gaining of insights on a larger scale, also including employees with lower educational levels. Again, a mixed methods approach should be applied to study both the effectiveness of the automated e-coach on stress, resilience, and reflection outcomes and to understand which design elements contribute to the effectiveness and why.

Specifically, future research can, for example, combine log data of the continuous biofeedback (eg, when the e-coach offers guidance to perform reflection-in-action and reflection-on-action) with the participant’s answers to the EMA questionnaires to study the output of the reflection process during moments that are in close occurrence to the stressful situation and during moderate levels of stress.

### Conclusions

The results of this study provide insights into the potential of automated e-coaching to guide employees during the reflection process for the purpose of resilience training. Most completers reported an improved energy balance and insights into their principal energy leaks and sources as well as effective strategies for improving their situation. The results indicate that an automated e-coach can guide employees during the reflection process on self-tracking data toward a deeper understanding of their situation and possible strategies to improve their situation. Design elements that stimulated the re-evaluation of situations and observation of trends stimulated the reflection process. It was more difficult to guide the employees via an automated e-coach to integrate the strategies into daily lives and reflect on whether the chosen strategies were the right ones. Future designs of the automated e-coach should make more and better use of persuasive features to support and motivate behavior change. Future research should focus on testing the effects on the reflection process by equipping the automated e-coach with more and improved persuasive features, as suggested above.

## Appendix

Multimedia Appendix 1 Description of the BringBalance app according to the consort guideline on reporting eHealth.

Multimedia Appendix 2 Post-test survey.

Multimedia Appendix 3 Interview scheme BringBalance.

Multimedia Appendix 4 Utility scores for the design elements of BringBalance per phase of reflection.

Multimedia Appendix 5 Summary table of stimulators and stagnating factors for reflection per phase of reflection.

## Abbreviations

BMS: Behavioural, Management and Social Sciences
BRS: Brief Resilience Scale
CONSORT: Consolidated Standards of Reporting Trials
EMA: ecological momentary assessment
HR: human resources
HRV: heart rate variability
PSS: Perceived Stress Scale
RQ: research question
TIIM: The Incredible Intervention Machine

## Data Availability

The data sets used and analyzed during this study are not publicly available because no consent was provided by the respondents before data collection took place. The data sets are available from the corresponding author on request.
