# Supplementary material for: How Reflective Automated e-Coaching Can Help Employees Improve Their Capacity for Resilience: Mixed Methods Study
Source: JMIR Hum Factors. 2023 Mar 10;10:e34331. doi: 10.2196/34331 (PMC10039404; doi:10.2196/34331)
Supplement: Multimedia Appendix 1 [file humanfactors_v10i1e34331_app1.docx]

## Multimedia Appendix 1 – Description of the BringBalance app according to the CONSORT guideline on reporting eHealth

|  | *Subitem CONSORT reporting eHealth guidelines [50]* |
| --- | --- |
| i | *Mention names, credential, affiliations of the developers, sponsors, and owners (if authors/evaluators are owners or developer of the software, this needs to be declared in a “Conflict of interest” section).*  **Developers BringBalance:**  Ewold de Maar, MSc, De Maar Training & Advies, Glimmen, The Netherlands  Aniek Lentferink, MSc, Psychology, Health, & Technology, University of Twente, Enschede, The Netherlands & Marian van Os Centre for Entrepreneurship, Hanze University of Applied Sciences, Groningen, The Netherlands  Prof. dr. Lisette van Gemert-Pijnen, Psychology, Health, & Technology, University of Twente, Enschede, The Netherlands  Dr. Hugo Velthuijsen, Marian van Os Centre for Entrepreneurship, Hanze University of Applied Sciences, Groningen, The Netherlands  Dr. Hilbrand Oldenhuis, Marian van Os Centre for Entrepreneurship, Hanze University of Applied Sciences, Groningen, The Netherlands  **Developers The Incredible Intervention Machine:**  Behavioural, Management and Social Sciences Lab (BMS Lab), University of Twente, Enschede, The Netherlands  **Sponsors:**  De Maar Training & Advies  Menzis  Hanze University of Applied Sciences  University of Twente  **Owner content BringBalance programme:** Ewold de Maar, MSc, De Maar Training & Advies, Glimmen, The Netherlands  **Owner the Incredible Intervention Machine:**  Behavioural, Management and Social Sciences Lab (BMS Lab), University of Twente, Enschede, The Netherlands |
| ii | *Describe the history/development process of the application and previous formative evaluations (e.g., focus groups, usability testing), as these will have an impact on adoption/use rates and help with interpreting results.*  The content of the BringBalance app is based on the face-to-face coaching programme *Working on Resilience* by De Maar Training & Advies. Results from a pilot study on this face-to-face coaching programme indicated positive effects on stress reduction [33].  In addition, the prototype version of the BringBalance app is developed following the CeHRes Roadmap, a roadmap for the development of eHealth with a high focus on involving all important stakeholders and the principles from business modeling [64]. Earlier research included a scoping review to identify critical success factors for self-tracking and persuasive eCoaching [27] and a needs assessment among employees and HR advisors by means of interviews [34] and focus groups among all identified key stakeholders using a business modelling approach [26]. The identified key stakeholders were employees, employers, representative councils within organisations, HR advisors, product owners, company doctors and business analysts [26].  This study is part of the design phase, the third phase, of the CeHRes Roadmap and includes testing a first prototype of the BringBalance programme using an existing app: The Incredible Intervention Machine (TIIM) app. Results can lead to the revision of earlier identified values and requirements in the first two phases of the CeHRes roadmap, namely the contextual inquiry and value specification phase, or the discovery of new values and requirements to improve the current design. |
| iii | *Revisions and updating. Clearly mention the date and/or version number of the application/intervention (and comparator, if applicable) evaluated, or describe whether the intervention underwent major changes during the evaluation process, or whether the development and/or content was “frozen” during the trial. Describe dynamic components such as news feeds or changing content which may have an impact on the replicability of the intervention.*  In this study, a first prototype of the BringBalance app was tested (version October 2018). The BringBalance app is in technical readiness level three “Proof of concept” [51, 52]. The applications and the content of the BringBalance programme in the TIIM app were frozen during the study. The applications did not make use of dynamic components other than the personal self-tracking data and the biofeedback related components from the Bringbalance app. |
| iv | *Provide information on quality assurance methods to ensure accuracy and quality of information provided, if applicable.*  The BringBalance app was pretested by two persons before the app was used in the study. This resulted in improving the navigation in the app as some elements were unintendently missed (such as short clips with the BringBalance techniques), clearifation of unclear spoken or written text, and usability adjustments such as decreasing the number of notifications by the app, enabling to check certain design elements more than once, and to set reminders for self-reporting of stress and resilience on natural moments during the day (end of the morning, afternoon, and evening) instead of 10:00 h, 14:00 h ect.  In addition, the developers of the Sense-IT app and the TIIM app were available for assistance during the experience of difficulties by the users of the apps. |
| v | *Ensure replicability by publishing the source code (preferably as open source), and/or providing screenshots/screen-capture video, and/or providing flowcharts of the algorithms used.*  *Replicability (i.e., other researchers should in principle be able to replicate the study) is a hallmark of scientific reporting.*  The source code is not open source. Screenshots of the BringBalance programme via the TIIM app are included in the article (see Figure 1 and 2). |
| vi | *Digital preservation: Provide the URL of the application, but as the intervention is likely to change or disappear over the course of the years, also make sure the intervention is archived (Internet Archive, webcitation.org, and/or publishing the source code or screenshots/videos alongside the article). As pages behind login screens cannot be archived, consider creating demo pages which are accessible without login.*  Screenshots of the BringBalance programme via the TIIM app are included in the article (see Figure 1 and 2). |
| vii | *Access: Describe how participants accessed the application, in what setting/context, if they had to pay (or were paid) or not, whether they had to be a member of specific group. If known, describe how participants gained “access to the platform and Internet”. To ensure access for editors/reviewers/readers, consider providing a “backdoor” login account or demo mode for reviewers/readers to explore the application (also important for archiving purposes, see vi).*  Employees of a software company in the east of the Netherlands could opt-in. They were recruited via the HR department of the company. The HR department informed the potential participants about the objectives of the study, the BringBalance app, data collection and management, and the amount of effort that was requested for employee participation. Employees willing to participate were asked to fill in an online questionnaire with the validated Dutch-version of the Perceived Stress Scale (PSS) [53-55] and an informed consent form. The inclusion criteria for participation was a score above 14 on the PSS, indicating a higher than average perceived level of stress [56, 57]. This inclusion criteria was based on earlier studies performed by the authors [26, 58] that showed employees with a certain level of stress tend to have a higher motivation to complete the intervention due to a higher expected benefit in comparison to employees with lower stress levels. Finally, participants needed to own an Android (version 5.0 or higher) or iOS (version 10.0 or higher) smartphone.  A total of 45 participants filled in the questionnaire, a response rate of 13%. Since fifteen HRV sensors were available, thirty participants were invited to join-in either one of two sessions: November 2018 (n=15) or January 2019 (n=15). Participation in the study was voluntary. Two days were planned to help the participants install the TIIM app and the InnerBalance Trainer app and to connect their InnerBalance trainer with the app. The instructions were also provided via email to the participants. Participants used their own smartphone and received an InnerBalance trainer during the study period. |
| viii | *Describe mode of delivery, features/functionalities/components of the intervention and comparator, and the theoretical framework used to design them (instructional strategy, behaviour change techniques, persuasive features, etc., see e.g., for terminology). This includes an in-depth description of the content (including where it is coming from and who developed it), “whether [and how] it is tailored to individual circumstances and allows users to track their progress and receive feedback”. This also includes a description of communication delivery channels and – if computer-mediated communication is a component – whether communication was synchronous or asynchronous. It also includes information on presentation strategies, including page design principles, average amount of text on pages, presence of hyperlinks to other resources etc.*  A concise description of the BringBalance app can be found at the end of the introduction of the article.  A schematic overview of the programme can be found in the table below. The BringBalance app is a prototype that consists of two apps: (1) TIIM app including the full content of the programme and (2) the Inner Balance app (HeartMath Institute) for the receiving biofeedback via the Inner Balance sensor during the practicing of the BringBalance Techniques (see Table). Below, we will describe some elements of the app in more detail. The theoretical framework used to build the content of the BringBalance app included literature on persuasive features, coaching techniques, reflection, and the earlier performed studies during the needs assessment [26, 27, 34]. The literature on persuasive features included the Persuasive System Design Model [22], the Fogg behavioural model [44], guidelines for persuasive interfaces [45], and earlier theory on effective persuasive elements for reflection [17]. Persuasive elements used in the app were for example *self-monitoring* using the EnergyBalance questionnaires (see described below), *reduction* by cutting down the reflection process in smaller steps, *personalisation* by helping them choose their own strategies to apply in daily life, and *rehearsal* by guiding the user through the practicing of the BringBalance techniques using short clips with instructions, guiding questions and biofeedback via the Inner Balance sensor.  The literature on coaching techniques and reflection included the reflective coaching model [15], levels of reflection [35], reflection via technology [10, 12, 24, 36, 37], 4G scheme [38], circumplex model of affect [42], implementation intentions [40], the four levels of evaluation [41], cognitive coaching [39], and the growth model [43].  **Table.** Content of the BringBalance programme   \| Phase \| Duration \| What? \| \| --- \| --- \| --- \| \| Phase 1 – Identification \| –2 weeks \| **Three times per day:**   - Filling in the EnergyBalance questionnaire (during the weekend once daily)   **Once daily:**   - Reflecting on the measurements of the day before   **End of phase 1:**   - Choosing the three most important energy sources and leaks   ***Result: Self-tracking data on the EnergyBalance for comparison with phase 3, list of energy sources and leaks and top three most important sources and leaks.*** \| \| Phase 2 – Strategy generation \| 2 weeks \| **Every Monday, Wednesday and Friday:**   - Learning a new BringBalance technique   **The day after the introduction of the technique:**   - Practicing the BringBalance technique with the Inner Balance Trainer   **End of phase 2:**   - Choosing strategies for their three most important energy sources and –leaks - Setting implementation intentions and reminders for phase 3   ***Result: Strategies were chosen for the top three energy sources and leaks, implementation intentions were set including the strategies for the energy sources and leaks, reminders were set with the implementation intentions*** \| \| Phase 3 - Experimenting \| –2 weeks \| **Daily:**   - Receiving reminders at chosen moments with their implementation intentions - Experimenting with the chosen strategies (optional: using the Inner Balance sensor) according to implementation intentions - Evaluating the strategy with a strategy evaluation form after experimenting with a strategy - Filling in the EnergyBalance questionnaire once daily   ***Result: Data on the evaluation of the strategies, self-tracking data on the EnergyBalance for comparison with phase 1*** \| \| Phase 4 – Evaluation \| 1 day \| **At the end of the programme:**   - Receiving the data collected in phase 3 via visualisations in tables and graphs - Evaluating if the strategies helped to prevent or resolve energy leaks and helped to make more use of energy sources. - Evaluating if the energy balance improved. - Advice on how to continue working on their energy balance after completion of the programme   ***Result: Final reflection on the strategies and energy balance and advice on how to continue working on their energy balance*** \|   **Examples of EMA questionnaires in the BringBalance app**  The advice given by Burke and Shiffman [21] was applied during the development of EMA questionnaires. Information given here on the EMA-questionnaires are based on the checklist provided in the article by Van Berkel and colleagues [63]:   - Inter-notification time: due to the many different steps in the BringBalance programme, the inter-notification time was different each phase and sometimes even per day. For example, the EnergyBalance questionnaires (see below) were send at 12:00 h, 17:00 h, and 22:00 h on workdays and at 15:00 h on weekend days. Participants were instructed to allow the sending of notifications via the TIIM app. Participants received a notification whenever a new module was available in the app. At the end of phase 2, participants were able to set their own timing of reminders with the implementation intentions during phase 3. - Notification expiry: Notifications of the EnergyBalance questionnaire expired when a new EnergyBalance questionnaire became available. Most notifications did not expire during the study period. Some non-mandatory modules disappeared after 1 day to secure chaos in available modules in the app. - Inquiry limit: the number of notifications could vary between 1-8 notifications per day. The maximum notifications were received at the end of phase 2 when participants had to choose their strategies, and set implementation intentions and reminders with those implementation intentions. - Participants did not receive a reward for their participation. - EMA question: See below. - Rich media collection: The input from participants on the EMA questionnaires were dropdown menu, text, yes/no answers or scores on a scale from 1-10. - Validated questionnaire adaptation: EMA questions were not validated questionnaires. The theoretical framework described above was input for developing the questions. The questions were pre-tested with two persons.   *EMA questions EnergyBalance (phase 1):*   1. What was your most important energy source of this morning/afternoon/evening? (text entry) 2. How energetic did you feel on a scale from 1-10? 3. Did you feel positive, neutral or negative? 4. What was your most important energy leak of this morning/afternoon/evening? (text entry) 5. How energetic did you feel on a scale from 1-10? 6. Did you feel positive, neutral or negative?   *EMA questions practicing BringBalance techniques (phase 2):*   1. What has the Neutral practicing day brought you?    1. Less balance, I feel less energetic    2. Just as much balance as usual, I feel the same as usual    3. More balance, I feel more energetic 2. What is the most important lesson you have learned from the Neutral technique? (text entry) 3. How could you integrate the Neutral in your daily life? (text entry)   Think of situations in which the Neutral might be useful, during what moments of the day and how long you should use the Neutral to achieve the best results.  *EMA questions strategy evaluation form (phase 3):*   1. Which strategy did you try out? (dropdown menu) 2. On a scale from 1-10: To what extent has the strategy helped you with this energy leak? 3. Do you feel more energetic? (Yes/No) 4. Do you feel more pleasant? (Yes/No) 5. If you have used the Inner Balance sensor, please note your coherencescore over here: 6. On a scale from 1-10: How easy did you find it to complete the strategy on a scale from 1-10? 7. On a scale from 1-10: How relevant was it to perform the strategy in this specific situation? 8. On a scale from 1-10: How much did you enjoyed performing the strategy? 9. Make a short note about your experience with performing the strategy: (text entry)   *For example, make notes about:*  *- What has the strategy brought you?*  *- What factors have worked against you (barriers)?*  *- What factors have stimulated you?*  *- How did people around you react?*  *EMA questions evaluating the strategies (phase 4):*   1. Presentation of collected data in a graph and table of the strategy evaluation forms in phase 3 on that specific strategy. 2. Why couldn't you give a 10 to the question "To what extent has the strategy helped you with this energy leak?" (text entry) 3. What does it take to make it a 10? (text entry) 4. What is the most positive aspect that you experienced during performing this strategy in situations related to this energy leaks? (text entry) 5. What is the most negative aspect that you experienced during performing this strategy in situations related to this energy leaks? (text entry) 6. What is the most important lesson that you have learned by applying the strategy in situations related to this energy leaks? (text entry) 7. What factors have stimulated you to perform the strategy? Can you make more use of such factors in the future? (text entry) 8. What factors have worked against you to perform the strategy? Can you eliminator those factors in the future? (text entry) 9. On the basis of the answers given until now, do you feel the need to adjust the strategy of this energy leaks? (Yes/No)   The BringBalance Techniques based on exercises from the HeartMath Institute [48]:  Every technique started with a breathing exercise, which could then be followed by evoking a positive emotion or by a framing exercise including: (1) framing a future event positively to reduce stress responses towards this event, (2) taking a moment to frame a current event in order to improve performance during this event, or (3) reframing a past event positively to reduce stress responses because of this event.  Persuasive design elements in Bringbalance  Figure 5 and 6 shows what persuasive design elements were present per phase of reflection in BringBalance.    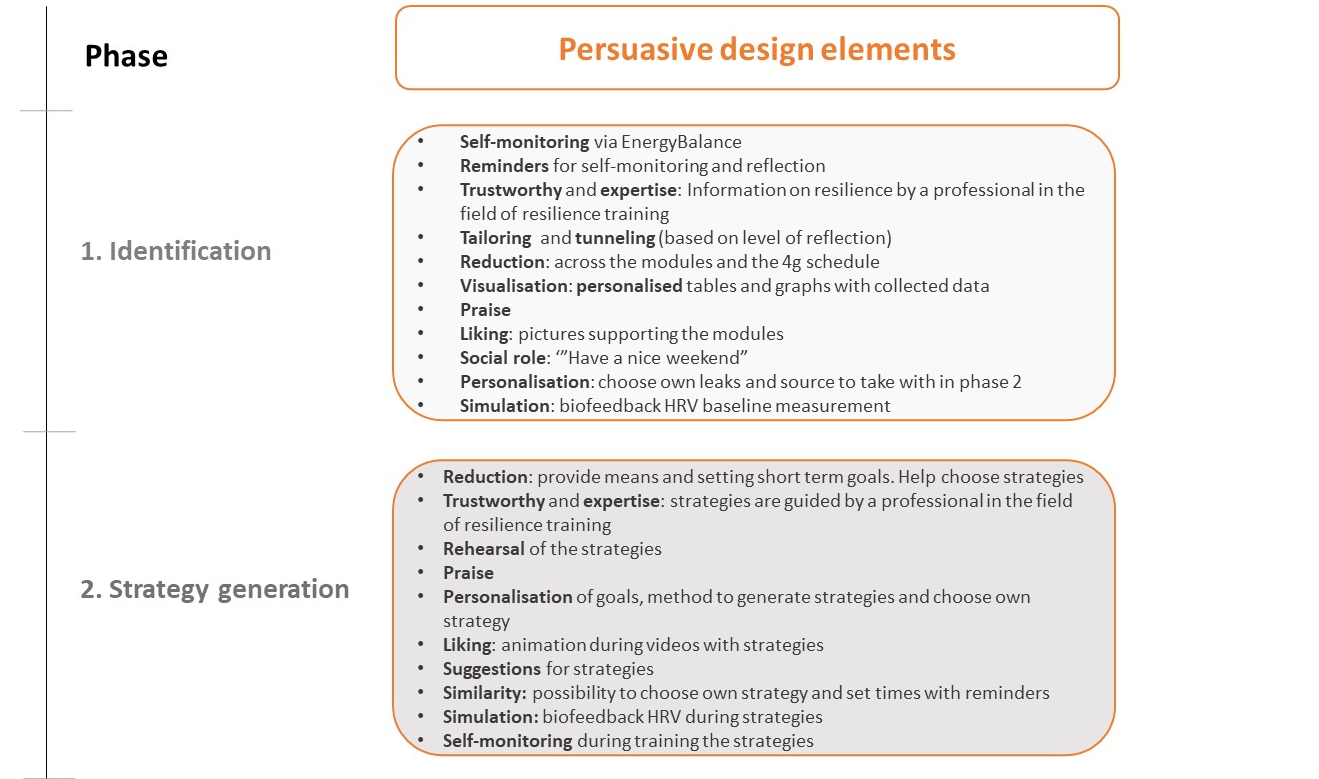  **Figure 5.** Persuasive design elements in BringBalance phase 1 and 2  **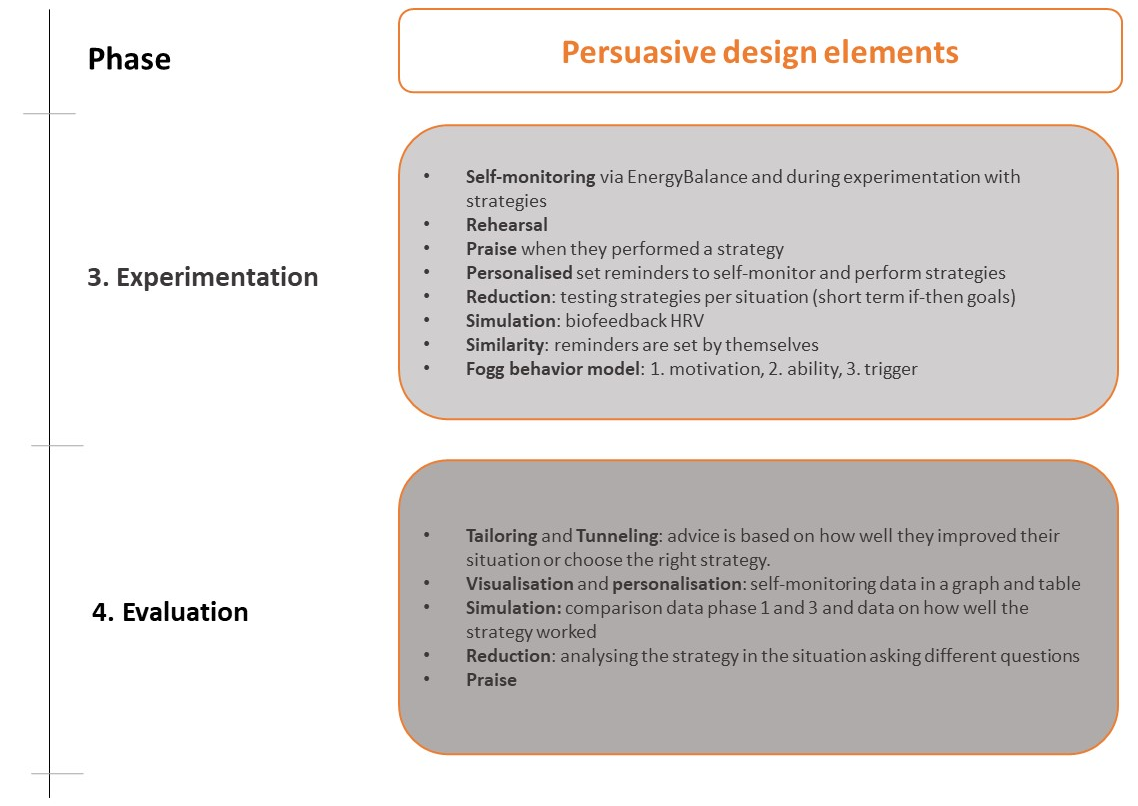**  **Figure 6.** Persuasive design elements of BringBalance in phase 3 and 4 of reflection |
| ix | *Describe use parameters (e.g., intended “doses” and optimal timing for use). Clarify describe what instructions or recommendations were given to the user, for example, regarding timing, frequency, heaviness of use, if any, or was the intervention used ad libitum*  Participants were instructed to use the BringBalance application daily during a study period of six weeks. About 15 minutes of time was asked from the participants on a daily basis with the exception of the weekend days, during which the app was used less intensively. Whenever they received a notification form the app, they were instructed to act upon the notification by checking out the available module in the TIIM app. |
| x | *Clarify the level of human involvement (care providers or health professionals, also technical assistance) in the e-intervention or as co-intervention. Detail number and expertise of professionals involved, if any, as well as “type of assistance offered, the timing and frequency of the support, how it is initiated, and the medium by which the assistance is delivered”. It may be necessary to distinguish between the level of human involvement required for the trial, and the level of human involvement required for a routine application outside of an RCT setting (discuss under item 21 – generalizability).*  The experiment leader (AL) was only involved during the intake of the participant and for problem solving during the study period. A week before the study period, the experiment leader was available on location for two days to help install the apps and to answer questions. In addition, before the start of the study, the participant received an instruction video and written instructions including a description of the BringBalance app, the installation of the apps, how to interact with the app in practice and instruction were provided on possible difficulties when using the app. Instructions on how to interact with the app included, among others, the advice to not skip modules as that could affect other steps in the BringBalance programme, not to mute notifications of the app, and to be aware about not skipping clips in the app (as they could be easily missed). Instructions on possible difficulties included what to do when they do not receive a notification by the app and what to do when the clips do not include sound.  Assistance was available on request during the experiment by mail or phone by the experiment leader. The intervention was executed without human involvement. |
| xi | *Report any prompts/reminders used: Clarify if there were prompts (letters, emails, phone calls, SMS) to use the application, what triggered them, frequency, etc. It may be necessary to distinguish between the level of prompts/reminders required for the trial, and the level of prompts/reminders for a routine application outside of an RCT setting (discuss under item 21 – generalizability).*  Users received reminders whenever a module became available in the app. Most of the reminders were set by the experiment leader. The reminders in phase 3 with the user’s personal set implementation intentions were set personally. See additional information about notifications described above in the section about the EMA questionnaires. |
| xii | *Describe any co-interventions (including training/support): Clearly state any “interventions that are provided in addition to the targeted eHealth intervention”, as eHealth intervention may not be designed as standalone intervention. This includes training sessions and support. It may be necessary to distinguish between the level of training required for the trial, and the level of training for a routine application outside of an RCT setting (discuss under item 21 – generalizability).*  The prototype of the BringBalance app consisted of the TIIM application in combination with the Inner Balance application. |
