# Supplementary material for: How Reflective Automated e-Coaching Can Help Employees Improve Their Capacity for Resilience: Mixed Methods Study
Source: JMIR Hum Factors. 2023 Mar 10;10:e34331. doi: 10.2196/34331 (PMC10039404; doi:10.2196/34331)
Supplement: Multimedia Appendix 2 [file humanfactors_v10i1e34331_app2.docx]

## Multimedia Appendix 2 – Post-test survey

BringBalance – English translation

**BringBalance questionnaire**
 
This questionnaire consists of the following parts:

- Your experiences with BringBalance
- Your experiences with the elements in the BringBalance programme
- The Perceived Stress Scale
- The Brief Resilience Scale

It will take about 30 minutes to complete the questionnaire.

Q1 Please, fill in your name:

________________________________________________________________

**Part 1 – Your experiences with BringBalance**

Firstly, we would like to ask you some questions about your experiences with the BringBalance program in general.

Q2. The following statements are about your perceived effect of the automated eCoach in the BringBalance app.

Please indicate to what extent you agree with the statements.

|  | Strongly disagree | Disagree | Neutral | Agree | Strongly agree |
| --- | --- | --- | --- | --- | --- |
| 1. The eCoach has given me a clear overview of my most important energy leaks and energy sources. |  |  |  |  |  |
| 2. Thanks to the eCoach, I know what I could do in future to prevent or resolve energy leaks. |  |  |  |  |  |
| 3. Thanks to the eCoach, I know what I could do in the future to take more advantage of my energy sources. |  |  |  |  |  |

Q3. The following statements are about the degree to which the BringBalance programme motivated you. Please indicate to what extent you agree with the statements.

|  | Strongly disagree | Disagree | Neutral | Agree | Strongly agree |
| --- | --- | --- | --- | --- | --- |
| 1. The BringBalance programme motivated me to reflect on my energy leaks and sources. |  |  |  |  |  |
| 2. The BringBalance programme motivated me to reflect on the chosen strategies for my energy leaks and sources. |  |  |  |  |  |

Q4. On a scale of 1-10: What score would you give the BringBalance programme in general?

ₒ 1 ₒ 2 ₒ 3 ₒ 4 ₒ 5 ₒ 6 ₒ 7 ₒ 8 ₒ 9 ₒ 10

Q5. Briefly describe why you gave this rating:

_________________________________________________________________________

Q6. Please indicate on a scale from 1-10: How much did you learn from the BringBalance program?

ₒ 1 ₒ 2 ₒ 3 ₒ 4 ₒ 5 ₒ 6 ₒ 7 ₒ 8 ₒ 9 ₒ 10

Q7. Describe here the three main things you have learned from BringBalance:

________________________________________________________________

Q8. Indicate on a scale of 1-10: To what extent did the BringBalance program appeal to you?

ₒ 1 ₒ 2 ₒ 3 ₒ 4 ₒ 5 ₒ 6 ₒ 7 ₒ 8 ₒ 9 ₒ 10

Q9. On a scale of 1-10: How useful did you find it to complete the BringBalance program?

ₒ 1 ₒ 2 ₒ 3 ₒ 4 ₒ 5 ₒ 6 ₒ 7 ₒ 8 ₒ 9 ₒ 10

Q10. On a scale from 1-10: How would you rate the usability of the BringBalance program?

ₒ 1 ₒ 2 ₒ 3 ₒ 4 ₒ 5 ₒ 6 ₒ 7 ₒ 8 ₒ 9 ₒ 10

Q11. On a scale of 1-10: How easy was it to integrate the BringBalance program into your daily life?

ₒ 1 ₒ 2 ₒ 3 ₒ 4 ₒ 5 ₒ 6 ₒ 7 ₒ 8 ₒ 9 ₒ 10

Q12. Would you recommend the BringBalance program to a colleague?

- Yes
- No

Q13.   Before the start you described your expectations of the BringBalance program. We have emailed you this expectation, together with the link to this questionnaire. On a scale of 1-10: To what extent did BringBalance meet this expectation?

ₒ 1 ₒ 2 ₒ 3 ₒ 4 ₒ 5 ₒ 6 ₒ 7 ₒ 8 ₒ 9 ₒ 10

Q14.  Briefly describe why you gave this rating:

________________________________________________________________

**Part 3 – Elements from the BringBalance programme**

You will see a number of elements from the BringBalance program. We are curious to what extent these elements have helped you in reflecting on situations related to your energy balance and determining and evaluating strategies for your energy leaks and sources. If you have not fully finished the BringBalance program, fill in the questions for the elements that you have gone through.

If you want to fully view a module from the BringBalance program again, please use the links below:

Phase 1: <https://app.tech4people-apps.bms.utwente.nl/preview/nuJvh/517>

Phase 2: <https://app.tech4people-apps.bms.utwente.nl/preview/YnIDY/475>

Phase 3: <https://app.tech4people-apps.bms.utwente.nl/preview/NMwON/462>

Phase 4: <https://app.tech4people-apps.bms.utwente.nl/preview/A02dL/528>

The questions are asked per phase. The last question contains space for comments about elements from that phase.

Q15 **Phase 1. The EnergyBalance**

You received the Energy Balance three times a day with the following questions:

*- What was your most important energy leak or source of the past half day?*

*- How energetic did you feel on a scale of 1-10?*

*- Did you feel pleasant, neutral or unpleasant at that time?*

***Indicate on a scale of 1-5 (1 = not at all, 5 = very much):***

***To what extent has this element helped you gain insight into your energy leaks and energy sources?***

ₒ 1 ₒ 2 ₒ 3 ₒ 4 ₒ 5

Q16 **Completing the Energy Balance three times a day was:**

- Not enough to get a clear overview of my energy leaks and sources during the day.
- Just right to get a clear overview of my energy leaks and resources during the day.
- Too often to get a clear overview of my energy leaks and resources during the day.

Q17 **Phase 1. Look back on the previous day - Graph.**

***Indicate on a scale of 1-5 (1 = not at all, 5 = very much):***

***To what extent has the graph helped you gain insight into your energy leaks and energy sources?***

ₒ 1 ₒ 2 ₒ 3 ₒ 4 ₒ 5

Q18 **Phase 1. Look back on the previous day - Table.**

***Indicate on a scale of 1-5 (1 = not at all, 5 = very much):***

***To what extent has the table helped you gain insight into your energy leaks and energy sources?***

ₒ 1 ₒ 2 ₒ 3 ₒ 4 ₒ 5

Q19 **Phase 1. Look back on the previous day – 4G questions**

During the look back, you chose the most important energy leak and source of the previous day. There are a number of questions:

  - What happened exactly? / Where did it happen? / When did it happen? / Who was present?

  - What emotions did you experience during this situation?

  - What were your physical reactions in this situation?

  - What thoughts were you having at the time?

  - How would you describe your behaviour in this situation?

  - Describe your energy leak (source) in keywords.

     You may never have been asked these questions. Then choose 'not applicable' (n / a).
***Indicate on a scale of 1-5 (1 = not at all, 5 = very much):***

***To what extent has this element helped you gain insight into your energy leaks and energy sources?***

ₒ 1 ₒ 2 ₒ 3 ₒ 4 ₒ 5 ₒ n / a

Q20 **Phase 1. Look back on the previous day – personalisation of the questions**

Based on the following three categories, you were referred to all 4G questions as mentioned above (option 1 or 2) or you could immediately describe the energy leak or energy source in keywords (option 3):

1. *I look at the data but I can't give it any meaning.*
2. *On the basis of the graph and table I can indicate what and when something gave me energy and or cost me energy, but I find it difficult to clearly identify what exactly happened then.*
3. *When I see the graph and the table I can give a good interpretation of what was going on at the time, how I felt physically and emotionally and what my reaction was at the time.*

***Indicate on a scale of 1-5 (1 = not at all, 5 = very much):***

***To what extent has the choice between these options helped you gain insight into your energy leaks and energy sources?***

ₒ 1 ₒ 2 ₒ 3 ₒ 4 ₒ 5

Q21 **Phase 1. Top 3 energy leaks and sources**

During the last module of phase 1, you saw an overview of your energy leaks and sources. From this overview you chose the three most important energy leaks and energy sources.
***Indicate on a scale of 1-5 (1 = not at all, 5 = very much):***

***To what extent has this element helped you gain insight into your most important energy leaks and energy sources?***

ₒ 1 ₒ 2 ₒ 3 ₒ 4 ₒ 5

Q22 **Phase 1. Reminders to fill in the EnergyBalance questionnaire**During phase 1 you received reminders when a new EnergyBalance was ready for you and when you had not yet filled in the EnergyBalance questionnaire after a certain period.
***Indicate on a scale of 1-5 (1 = not at all, 5 = very much):***

***To what extent has this element helped you gain insight into your energy leaks and energy sources?***

ₒ 1 ₒ 2 ₒ 3 ₒ 4 ₒ 5

Q23 **Phase 1. Reminders to fill in the module ‘Look back on the previous day’**During phase 1 you received reminders when you could look back on the day before and when you hadn't looked back on the day before after a while.
***Indicate on a scale of 1-5 (1 = not at all, 5 = very much):***

***To what extent has this element helped you gain insight into your energy leaks and energy sources?***

ₒ 1 ₒ 2 ₒ 3 ₒ 4 ₒ 5

Q24 **Space for comments about the elements from phase 1.**

________________________________________________________________

________________________________________________________________

________________________________________________________________

________________________________________________________________

________________________________________________________________

Q25 **Phase 2. BringBalance techniques** In phase 2, you were introduced every other day to a new BringBalance technique, including videos:

  - Neutral

  - Shift

  - Preframe

  - Reframe

  - Flexframe

  - Zzleep

***Indicate on a scale of 1-5 (1 = not at all, 5 = very much):***

***To what extent has this element helped you determine appropriate strategies for your energy leaks and resources?***

ₒ 1 ₒ 2 ₒ 3 ₒ 4 ₒ 5

Q26 **Indicate on a scale of 1-5 (1 = not at all, 5 = very much): How clear did you find the animation videos?**

ₒ 1 ₒ 2 ₒ 3 ₒ 4 ₒ 5

Q27 **Indicate on a scale of 1-5 (1 = not at all, 5 = very much): How clear did you find the text in the modules with the BringBalance techniques?**

ₒ 1 ₒ 2 ₒ 3 ₒ 4 ₒ 5

Q28 **Indicate on a scale of 1-5 (1 = not at all, 5 = very much): To what extent did the animation films appeal to you?**

ₒ 1 ₒ 2 ₒ 3 ₒ 4 ₒ 5

Q29 **Please indicate on a scale of 1-5 (1 = not at all, 5 = very much): How easy was it for you to learn the BringBalance techniques?**

ₒ 1 ₒ 2 ₒ 3 ₒ 4 ₒ 5

Q30 **What did you think of the variation in BringBalance techniques you learned?**

- Too little variation
- Just enough variation
- Too much variation

Q31 **Use the space below if you want to report something about the BringBalance techniques:**

________________________________________________________________

________________________________________________________________

________________________________________________________________

________________________________________________________________

________________________________________________________________

Q32 **Phase 2. Train BringBalance techniques**

   Every next day, after you were introduced to a new technique, you trained the technique.

  You received reminders to practise. If you have not been able to practise, enter 'not applicable' (n / a).

***Indicate on a scale of 1-5 (1 = not at all, 5 = very much):***

***To what extent has this element helped you determine appropriate strategies for your energy leaks and resources?***

ₒ 1 ₒ 2 ₒ 3 ₒ 4 ₒ 5 ₒ n / a

Q33 **Phase 2. Evaluate BringBalance training days**

   At the end of the training day you were asked to evaluate the training with the technique.

  You received the following questions:

- What did the training day yield to you?
- What is the most important thing you learned from the technique?
- How could you integrate the technique into your daily life?

  If you have not been able to evaluate the training day, enter 'not applicable' (n / a).

***Indicate on a scale of 1-5 (1 = not at all, 5 = very much):***

***To what extent has this element helped you determine appropriate strategies for your energy leaks and resources?***

ₒ 1 ₒ 2 ₒ 3 ₒ 4 ₒ 5 ₒ n / a

Q34 **Phase 2. Techniques in your daily life**

   This module provided you with an overview of your answers to the evaluations of the BringBalance techniques.

  If you have not been able to evaluate the training day, enter 'not applicable' (n / a).

***Indicate on a scale of 1-5 (1 = not at all, 5 = very much):***

***To what extent has this advice helped you determine appropriate strategies for your energy leaks and resources?***

ₒ 1 ₒ 2 ₒ 3 ₒ 4 ₒ 5 ₒ n / a

Q35 **Phase 2. Inner Balance Trainer while learning the techniques**

   While practicing the above techniques, you automatically received information via the sensor about the effects of the technique on your physical state. During the exercise you received feedback in the form of colors: Red: moderate coherence, blue: good coherence, green: excellent coherence. In addition, you received a summary of the measurements during the exercise at the end.

If you have not been able to train the techniques with the Inner Balance Trainer, enter 'not applicable' (n / a).

***Indicate on a scale of 1-5 (1 = not at all, 5 = very much):***

***To what extent has this element helped you determine appropriate strategies for your energy leaks and resources?***

ₒ 1 ₒ 2 ₒ 3 ₒ 4 ₒ 5 ₒ n / a

Q36 **Did you find it useful to use the Inner Balance app while learning the BringBalance techniques?**

- Yes
- No

Q37 **Describe here why you believed the Inner Balance trainer was (not) useful during the learning of the BringBalance techniques:**

________________________________________________________________

________________________________________________________________

________________________________________________________________

________________________________________________________________

________________________________________________________________

Q38 **Phase 2. HRV measurements**

In phase 1, you were asked to use the Inner Balance Trainer several times to perform a measurement.

In phase 2 you were asked to perform a measurement with the Inner Balance Trainer during the Neutral technique.

You received the differences between the measurements in the module 'Your HRV measurements'.

   If you have not started this module, enter 'not applicable' (n / a).
***Indicate on a scale of 1-5 (1 = not at all, 5 = very much):***

***To what extent has this element helped you determine appropriate strategies for your energy leaks and resources?***

ₒ 1 ₒ 2 ₒ 3 ₒ 4 ₒ 5 ₒ n / a

Q39 **Phase 2. Determining the energy leak strategies - Options**

You could determine a strategy that matched your wishes and needs for closing your energy leaks.

The strategy could consist of one of the six BringBalance techniques, one of your energy sources or a self-devised strategy. You could choose from the following options to determine a strategy:

1. I already have an idea: With this module you can immediately write down a strategy for the energy leak in question.
2. I would like to take a look at the strategy database: In this module you will find an overview of the learned BringBalance techniques and you will receive advice on how to use the BringBalance techniques for your energy leaks.
3. I would like help from the eCoach: In this module you will be asked a number of questions. From this, suggestions for strategies follows.

***Indicate on a scale of 1-5 (1 = not at all, 5 = very much):***

***To what extent has the choice in these options helped you determine appropriate strategies for your energy leaks and resources?***

ₒ 1 ₒ 2 ₒ 3 ₒ 4 ₒ 5

Q40 **Phase 2. Determining the energy leak strategies - I already have an idea myself**

   When you chose the option 'I already have an idea myself', you independently determined a strategy for your energy leak.

    If you have not started this module, enter 'not applicable' (n / a).

***Indicate on a scale of 1-5 (1 = not at all, 5 = very bad):***

***To what extent has the 'I already have an idea' option helped you to determine appropriate strategies for your energy leaks?***

ₒ 1 ₒ 2 ₒ 3 ₒ 4 ₒ 5 ₒ n / a

Q41 **Phase 2. Determining the strategies for energy leaks - Strategy database**

   In the strategy database, you have found an overview of the taught BringBalance techniques and you received advice on how to use the BringBalance techniques for your energy leaks.

   An example:

  In the Neutral video you received the following advice: "For example, if you feel restless or rushed, the Neutral can calm you down and regain balance. However, if you feel lifeless or tired, the Neutral can activate you" .

   You were also advised to take a look at your list of energy sources for possible strategies.

    If you have not started this module, enter 'not applicable' (n / a).

***Indicate on a scale of 1-5 (1 = not at all, 5 = very bad):***

***To what extent has the 'Strategy database' option helped you to determine appropriate strategies for your energy leaks?***

ₒ 1 ₒ 2 ₒ 3 ₒ 4 ₒ 5 ₒ n / a

Q42 **Phase 2. Determining the energy leak strategies - Help from the eCoach**

   You were asked a number of questions in this module. Based on your answers, suggestions for strategies followed.

   An example:

  If you answered 'Yes' to the question *'Is it an energy leak by which you experience a lot of tension beforehand?'* then you received the following suggestion*: "Then we advise you to apply the Preframe exercise prior to this energy leak."*

   If you have not started this module, enter 'not applicable' (n / a).

***Indicate on a scale of 1-5 (1 = not at all, 5 = very much):***

***To what extent has the 'Help from the eCoach' option helped you to determine appropriate strategies for your energy leaks?***

ₒ 1 ₒ 2 ₒ 3 ₒ 4 ₒ 5 ₒ n / a

Q43 **Phase 2. Determining the energy leak strategies - Advice for a strategy.**

   If you were unable to determine a strategy using the modules 'strategy database' and 'help from the eCoach', you received the following advice:

*"In order to ensure that your body is not constantly under tension during the day due to this energy leak, it would be good to occasionally relax by using the Neutral technique. We advise you therefore to apply the Neutral technique for 3 minutes 3 times a day for this energy leak, for example before you go to work in the morning, during the lunch break and in the evening after work. "*

   Enter 'not applicable' (n / a) if you have not used this option.

***Indicate on a scale of 1-5 (1 = not at all, 5 = very much):***

***To what extent has this advice helped you determine appropriate strategies for your energy leaks and resources?***

ₒ 1 ₒ 2 ₒ 3 ₒ 4 ₒ 5 ₒ n / a

Q44 **Phase 2. Determining the strategies for your energy sources**

   In this module, you were first asked whether you could already use the energy source as a strategy or if some adjustment was needed. If that was the case, you were asked what it took to be able to use the source more often.

***Indicate on a scale of 1-5 (1 = not at all, 5 = very much):***

***To what extent has this element helped you determine appropriate strategies for your energy leaks and resources?***

ₒ 1 ₒ 2 ₒ 3 ₒ 4 ₒ 5

Q45 **Phase 2. Module 'More Zzleep'**

   In this module, you received your average score on the question 'How well did you sleep last night?' from the Energy Balances filled in in phase 1.

  The following was included in the module:

  In the Energy Balances you gave your sleep quality an average score of 6.2 on a scale from 1-10. Would you like to improve your sleep quality based on this?

   Enter 'not applicable' (n / a) if you have not completed the module 'More Zzleep?'. 
***Indicate on a scale of 1-5 (1 = not at all, 5 = very much):***

***To what extent has this element helped you determine appropriate strategies for your energy leaks and resources?***

ₒ 1 ₒ 2 ₒ 3 ₒ 4 ₒ 5 ₒ n / a

Q46 **Phase 2. Set goals for the strategies for your leaks and sources.**

  In this module you received guidance in setting up your personal goals.

 You received the following information: Set a goal for energy leak 1 and the associated strategy.

 a. Situation: During which moment should you apply the strategy (this could be your energy leak)?

 b. When: Do you have to apply the strategy BEFORE, AFTER or DURING the moment to achieve the desired effect or at a fixed time of the day or week?

 c. Strategy: What strategy will you apply?

 d. Duration: How long do you have to apply the strategy?

 Example: "Before (b) giving a presentation (a), I apply the PreFrame technique (c) for 5 minutes (d)".

***Indicate on a scale of 1-5 (1 = not at all, 5 = very much):***

***To what extent did this element help you experiment and evaluate whether the chosen strategy was the right one for your energy leak or source?***

ₒ 1 ₒ 2 ₒ 3 ₒ 4 ₒ 5

Q47 Space for comments on the elements in phase 2:

________________________________________________________________

________________________________________________________________

Q48 **Phase 3. Reminders with your personal goals**

   During phase 3, you received reminders with your personal goals at the times set by you. Enter 'not applicable' (n / a) if you have not set any reminders for phase 3.
***Indicate on a scale of 1-5 (1 = not at all, 5 = very much):***

***To what extent did this element help you experiment and evaluate whether the chosen strategy was the right one for your energy leak or source?***

ₒ 1 ₒ 2 ₒ 3 ₒ 4 ₒ 5 ₒ n / a

Q49 **Phase 3. Experiment with the strategies**

During the experimentation phase, you tested your own chosen strategies in daily life per energy leak or source.

***Indicate on a scale of 1-5 (1 = not at all, 5 = very much):***

***To what extent did testing the strategies in daily life help you evaluate whether the chosen strategy was the right one for your energy leak or source?***

ₒ 1 ₒ 2 ₒ 3 ₒ 4 ₒ 5

Q50 **Phase 3. Experiment with the BringBalance techniques**

   During the experimentation phase, you could test the BringBalance techniques as a strategy for an energy leak or energy source in everyday life.

Enter 'not applicable' (n / a) if you have not used the BringBalance techniques as a strategy.

***Indicate on a scale of 1-5 (1 = not at all, 5 = very much):***

***To what extent has testing the BringBalance strategies in daily life helped you to evaluate whether the chosen strategy was the right one for your energy leak or source?***

ₒ 1 ₒ 2 ₒ 3 ₒ 4 ₒ 5 ₒ n / a

Q51 **Phase 3. Using the Inner Balance training during experimentation with the BringBalance techniques**

  If you have linked the BringBalance techniques to your energy leak (s) or energy source (s), you could use the Inner Balance Trainer to receive feedback. The sensor automatically gave you information about how the strategy influenced your physical state. During the exercise you received feedback in the form of colors: Red: poor coherence, blue: moderate coherence, green: good coherence. In addition, you received a summary of the measurements during the exercise at the end.

   Enter 'not applicable' (N / A) if you have not used the Inner Balance Trainer while experimenting with strategies.

***Indicate on a scale of 1-5 (1 = not at all, 5 = very much):***

***To what extent did this element help you experiment and evaluate whether the chosen strategy was the right one for your energy leak or source?***

ₒ 1 ₒ 2 ₒ 3 ₒ 4 ₒ 5 ₒ n / a

Q52 **Phase 3. Experiment with your energy sources to prevent or resolve energy leaks.**

   During the experimentation phase, yo u could test your energy sources as strategies for your energy leaks in everyday life.

     Enter 'not applicable' (n / a) if you have not used your energy sources as a strategy.

***Indicate on a scale of 1-5 (1 = not at all, 5 = very much):***

***To what extent has testing the energy source strategies in daily life helped you evaluate whether the chosen strategy was the right one for your energy leak?***

ₒ 1 ₒ 2 ₒ 3 ₒ 4 ₒ 5 ₒ n / a

Q53 **Phase 3. Experiment with self-devised strategies.**

During the experimentation phase, you could test a self-devised strategy as a strategy for an energy leak in everyday life.

Enter 'not applicable' (N / A) if you have not used a self-devised strategy

***Indicate on a scale of 1-5 (1 = not at all, 5 = very much):***

***To what extent has testing the self-devised strategies in daily life helped you evaluate whether the chosen strategy was the right one for your energy leak?***

ₒ 1 ₒ 2 ₒ 3 ₒ 4 ₒ 5 ₒ n / a

Q54 **Phase 3. Experiment to make better use of your energy sources.**

   During the experimentation phase, you experimented in daily life with the strategies to make better use of your energy sources.

   Enter 'not applicable' (N / A) if you were not in the ability to experiment with the strategies for your sources.

***Indicate on a scale of 1-5 (1 = not at all, 5 = very much):***

***To what extent has testing the strategies for your energy sources in daily life helped you to evaluate whether the chosen strategy was the right one to make better use of your energy sources?***

ₒ 1 ₒ 2 ₒ 3 ₒ 4 ₒ 5 ₒ n / a

Q55 **Phase 3. Strategy evaluation forms**

After testing a strategy for an energy leak or source, you were asked to fill in the strategy evaluation form.

The form contained the following questions:

- Which strategy did you try out? (dropdown menu)
- On a scale from 1-10: To what extent has the strategy helped you with this energy leak?
- Do you feel more energetic? (Yes/No)
- Do you feel more pleasant? (Yes/No)
- If you have used the Inner Balance sensor, please note your coherencescore over here:
- On a scale from 1-10: How easy did you find it to complete the strategy on a scale from 1-10?
- On a scale from 1-10: How relevant was it to perform the strategy in this specific situation?
- On a scale from 1-10: How much did you enjoyed performing the strategy?
- Make a short note about your experience with performing the strategy: (text entry)

Choose 'not applicable' (n / a) if you have not been able to complete the strategy evaluation forms.
***Indicate on a scale of 1-5 (1 = not at all, 5 = very much):***

***To what extent did this element help you experiment and evaluate whether the chosen strategy was the right one for your energy leak or source?***

ₒ 1 ₒ 2 ₒ 3 ₒ 4 ₒ 5 ₒ n / a

Q56 **Phase 3. EnergyBalance**

   In phase 3 you filled in the energy balance again. This time only at the end of the day.

  Select 'not applicable' (n / a) if you have not been able to complete the EnergyBalance questionnaires in phase 3.
***Indicate on a scale of 1-5 (1 = not at all, 5 = very much):***

***To what extent did this element help you experiment and evaluate whether the chosen strategy was the right one for your energy leak or source?***

ₒ 1 ₒ 2 ₒ 3 ₒ 4 ₒ 5 ₒ n / a

Q57 **Phase 3. Additional questions in the EnergyBalance**

The EnergyBalance contained a number of additional questions compared to phase 1:

- Did you succeed in following-up all the personal goals of today?
- What goal(s) did you fail to achieve?
- Make a short note below what prevented you from following up on the personal goal(s)

Enter 'not applicable' if you have not been able to complete the EnergyBalance questionnaires.

***Indicate on a scale of 1-5 (1 = not at all, 5 = very much):***

***To what extent did this element help you experiment and evaluate whether the chosen strategy was the right one for your energy leak or source?***

ₒ 1 ₒ 2 ₒ 3 ₒ 4 ₒ 5 ₒ n / a

Q58 Space for comments on the elements in phase 3:

________________________________________________________________

________________________________________________________________

________________________________________________________________

________________________________________________________________

________________________________________________________________

Q59 **Phase 4. Evaluate strategies - Graph**

During the evaluation of the strategies in phase 4, you received the results of the strategy evaluation forms from phase 3 in a graph.

Enter 'not applicable' (N / A) if no results were shown for you.

***Indicate on a scale of 1-5 (1 = not at all, 5 = very much):***

***To what extent has this element helped you to evaluate whether the chosen strategy was the right one for your energy leak or source?***

ₒ 1 ₒ 2 ₒ 3 ₒ 4 ₒ 5 ₒ n / a

Q60 **Phase 4. Evaluate strategies - Table**

  During the evaluation of the strategies in phase 4, you received the results of the strategy evaluation forms from phase 3 in a table.

  Enter 'not applicable' (N / A) if no results were shown for you.

***Indicate on a scale of 1-5 (1 = not at all, 5 = very much):***

***To what extent has this element helped you to evaluate whether the chosen strategy was the right one for your energy leak or source?***

ₒ 1 ₒ 2 ₒ 3 ₒ 4 ₒ 5 ₒ n / a

Q61 **Phase 4. Evaluate strategies - Questions**

  During the evaluation of the strategies in phase 4, you received the results of the strategy evaluation forms were visualized for you in a graph and table.

   Then you were asked the following questions:

- Why couldn't you give a 10 to the question "To what extent has the strategy helped you with this energy leak?" (text entry)
- What does it take to make it a 10? (text entry)
- What is the most positive aspect that you experienced during performing this strategy in situations related to this energy leaks? (text entry)
- What is the most negative aspect that you experienced during performing this strategy in situations related to this energy leaks? (text entry)
- What is the most important lesson that you have learned by applying the strategy in situations related to this energy leaks? (text entry)
- What factors have stimulated you to perform the strategy? Can you make more use of such factors in the future? (text entry)
- What factors have worked against you to perform the strategy? Can you eliminator those factors in the future? (text entry)
- On the basis of the answers given until now, do you feel the need to adjust the strategy of this energy leaks? (Yes/No)

Enter 'not applicable' (N / A) if no results were shown for you.

***Indicate on a scale of 1-5 (1 = not at all, 5 = very much):***

***To what extent has this element helped you to evaluate whether the chosen strategy was the right one for your energy leak or source?***

ₒ 1 ₒ 2 ₒ 3 ₒ 4 ₒ 5 ₒ n / a

Q62 **Phase 4. Evaluate whether your energy balance has improved - graph**

   While evaluating whether your energy balance has improved, the results of the EnergyBalances in phases 1 and 3 were visualized in a graph.

  Then you were asked the following question: Has your energy balance improved in recent weeks?

Enter 'not applicable' (N / A) if no results were shown for you.

***Indicate on a scale of 1-5 (1 = not at all, 5 = very much):***

***To what extent has this element helped you to evaluate whether the chosen strategy was the right one for your energy leak or source?***

ₒ 1 ₒ 2 ₒ 3 ₒ 4 ₒ 5 ₒ n / a

Q63 **Phase 4. Evaluate whether your energy balance has improved - Table**

   While evaluating whether your energy balance has improved, the results of the EnergyBalances in phases 1 and 3 were visualized in a table.

  Then you were asked the following question: Has your energy balance improved in recent weeks?

Enter 'not applicable' (N / A) if no results were shown for you.

***Indicate on a scale of 1-5 (1 = not at all, 5 = very much):***

***To what extent has this element helped you to evaluate whether the chosen strategy was the right one for your energy leak or source?***

ₒ 1 ₒ 2 ₒ 3 ₒ 4 ₒ 5 ₒ n / a

Q64 **Phase 4. Final advice**

 Based on your answers to the questions below, you received a final suggestion:

 - Has your energy balance improved in recent weeks?

 - If not, do you feel that the insights and strategies have brought you something?

The last suggestion could be one of these three options:

1. We are very sorry to hear that the BringBalance did not provide you with insights and / or useful strategies. Your situation may require a different approach. Is working on stress and resilience via an app really what you need? Talk to someone and look for other solutions.
2. You have gained more insight into your energy balance and / or strategies that can help! How nice! Improving the energy balance is not done overnight. The first important step is to gain insight. Without insight into your energy leaks and sources, you don't know where to start. The targeted use of strategies in order to improve your energy balance is the next step. Good that you have also gained some more knowledge about that. Keep looking for your personal energy leaks and resources and don't forget your learned strategies!
3. An improved energy balance! How nice that you have felt more energetic and pleasant in recent weeks. Keep looking for your personal energy leaks and resources and don't forget your learned strategies!

 Enter 'not applicable' (N / A) if you did not received a final advice.

***Indicate on a scale of 1-5 (1 = not at all, 5 = very much):***

***To what extent has this element helped you to understand what you could do in the future to improve your energy balance?***

ₒ 1 ₒ 2 ₒ 3 ₒ 4 ₒ 5 ₒ n / a

Q65 Space for comments about the elements in phase 4:

________________________________________________________________

________________________________________________________________

________________________________________________________________

________________________________________________________________

________________________________________________________________

**Part 4 – Brief Resilience Scale**

Q66 **The Brief Resilience Scale**

Again, we ask you to fill in the Brief Resilience Scale and Perceived Stress Scale so that we can compare the post-test scores with the scores of the scales prior to the start of BringBalance.

  Six statements are presented. For each statement, indicate to what extent you agree with the statement.

|  | Strongly disagree | Disagree | Neutral | Agree | Strongly agree |
| --- | --- | --- | --- | --- | --- |
| 1. I tend to bounce back quickly after hard times. |  |  |  |  |  |
| 2. I have a hard time making it through stressful events. |  |  |  |  |  |
| 3. It does not take me long to recover from a stressful event. |  |  |  |  |  |
| 4. It is hard for me to snap back when something bad happens. |  |  |  |  |  |
| 5. I usually come through difficult times with little trouble. |  |  |  |  |  |
| 6. I tend to take a long time to get over set-backs in my life. |  |  |  |  |  |

**Part 5 – Perceived Stress Scale**

Q67 **The Perceived Stress Scale**    

The 10 questions in this scale ask you about your feelings and thoughts during the last month. In each case, you will be asked to indicate, by checking the box, how often you felt or thought a certain way.

|  | Never | Almost never | Sometimes | Fairly often | Very often |  |
| --- | --- | --- | --- | --- | --- | --- |
| 1. In the last month, how often have you been upset because of something that happened unexpectedly? |  |  |  |  |  |  |
| 2. In the last month, how often have you felt that you were unable to control the important things in your life? |  |  |  |  |  |  |
| 3. In the last month, how often have you felt nervous and “stressed”? |  |  |  |  |  |  |
| 4. In the last month, how often have you felt confident about your ability to handle your personal problems? |  |  |  |  |  |  |
| 5. In the last month, how often have you felt that things were going your way? |  |  |  |  |  |  |
| 6. In the last month, how often have you found that you could not cope with all the things that you had to do? |  |  |  |  |  |  |
| 7. In the last month, how often have you been able to control irritations in your life? |  |  |  |  |  |  |
| 8. In the last month, how often have you felt that you were on top of things? |  |  |  |  |  |  |
| 9. In the last month, how often have you been angered because of things that were outside of your control? |  |  |  |  |  |  |
| 10. In the last month, how often have you felt difficulties were piling up so high that you could not overcome them? |  |  |  |  |  |  |

Thank you for filling in this questionnaire!
