# Supplementary material for: How Reflective Automated e-Coaching Can Help Employees Improve Their Capacity for Resilience: Mixed Methods Study
Source: JMIR Hum Factors. 2023 Mar 10;10:e34331. doi: 10.2196/34331 (PMC10039404; doi:10.2196/34331)
Supplement: Multimedia Appendix 3 [file humanfactors_v10i1e34331_app3.docx]

## Multimedia Appendix 3 – Interview scheme BringBalance

| Topic | Quesions | Remarks |
| --- | --- | --- |
| Introduction | 1. Goals of the study |  |
|  | 1. Aks permission for recording |  |
| Experience in general | 1. How did you experience the BringBalance programme? |  |
|  | 1. 2. What has the BringBalance program brought you? And what about stress and resilience? |  |
|  | 1. 3. Have there been any particularities in the past six weeks that influenced the use of the BringBalance? |  |
| Usability | 1. 1. From log data: you have/have not used these modules. Would you please explain why this was so? 2. What did you find difficult? And what was that about? 3. What did you find easy? And what was that about?   Examples? | Results of logdata per participant will be used during this question |
| Grade | 1. 1. You have rated the app with the grade … + short explanation.   Example? |  |
| Insights in energyleaks and -sources | 1. To what extent has the app helped you gain insight into your most important energy leaks and energy sources?   Reflection:   - Collecting of - Looking back on - Awareness - Understanding of | The score given in the survey on the gaining of insights will be used during this question |
|  | 1. Did you gain new insights while using BringBalance? |  |
|  | 1. Which element of the app helped you the most?  - What have you learned? - Could you give an example of how this element has helped you?   *Relevant elements in BringBalance:*   - *Movie EnergyBalance* - *Energy Balance 3 x daily* - *Looking back on yesterday* - *Determine top 3* | The score given in the survey on the utility of elements for reflection will be used during this question |
|  | 1. Are there any other elements of the app that helped you with that?  - What have you learned? - Could you give an example of how this element has helped you? | Whenever possible based on the score on the survey |
|  | 1. To what extent has the app challenged you to think more deeply about your energy leaks and sources than you would have done on your own? |  |
|  | 1. What else do you think the app could have done to help you understand your energy leaks and sources? |  |
| Insights in strategies to close energyleaks and make better use of energysources | 1. To what extent has the app helped you to gain insight into strategies that can help close the energy leaks and make better use of the energy resources?   Reflection:   - Understanding - Determining - Experimenting/Applying - Collecting of - Evaluating | The score given in the survey on the gaining of insights will be used during this question |
|  | 1. Did you gain new insights while using BringBalance? |  |
|  | 1. Which element of the app helped you the most?  - What have you learned? - Could you give an example of how this element has helped you?   *Relevant elements in BringBalance:*   - *Learning the six strategies* - *Biofeedback InnerBalance trainer (HRV measurements)* - *Techniques in your daily life* - *Determine the strategies for the energy leaks and sources (self, strategy database, help eCoach)* - *Setting goals* - *Reminders* - *Experimentation* - *Strategy evaluation questionnaire* - *EnergyBalance* - *Evaluate strategy per leak and source* - *Evaluate improved energy balance.* | The score given in the survey on the utility of elements for reflection will be used during this question |
|  | 1. Are there any other elements of the app that helped you with that?  - What have you learned? - Could you give an example of how this element has helped you? | Whenever possible based on the score on the survey |
|  | 1. To what extent has the app challenged you to think more deeply about possible strategies that can help you close energy leaks and make better use of energy sources than you would have done on your own? |  |
|  | 1. What else do you think the app could have done to help you understand strategies for your energy leaks and energy sources? |  |
| Reasons for dropping out (when applicable) | 1. What was the most important reason you stopped? 2. Could we somehow have prevented that? |  |
| Suggestions for improvement | 1. Do you have any suggestions for improvement on how you would like to be guided by the app in gaining insight into:  - The most important energy leaks and sources - Strategies that can help close the energy leaks and make better use of energy resources |  |
