# Supplementary material for: How Reflective Automated e-Coaching Can Help Employees Improve Their Capacity for Resilience: Mixed Methods Study
Source: JMIR Hum Factors. 2023 Mar 10;10:e34331. doi: 10.2196/34331 (PMC10039404; doi:10.2196/34331)
Supplement: Multimedia Appendix 4 [file humanfactors_v10i1e34331_app4.docx]

## Multimedia Appendix 4 – Utility scores for the design elements of BringBalance per phase of reflection

**Table.** The utility of the elements of BringBalance for the reflection process per phase.

| Element of the BringBalance app | Study non-completers (n=14)  M (SD)  n=number of responses to the question | Completers (n=14)  M (SD)  n=number of responses to the question | Number of participants filling in the questions in this phase  N=27 |
| --- | --- | --- | --- |
| Elements phase 1 (scale 1-5): ****did the element helped you to gain insights into energy leaks and sources?**** |  |  |  |
| EnergyBalance | 3.5 (0.7)  n=13 | 3.6 (0.8)  n=14 | 3.6 (0.8)  n=27 |
| EnergyBalance: Filling in the EnergyBalance three times per day was: too infrequent, just enough, too often | Too infrequent =0%  Just enough = 23%  Too often = 77%  n=13 | Too infrequent = 14%  Just enough = 50%  Too often = 36%  n=14 | Too infrequent = 7%  Just enough = 37%  Too often = 56%  n=27 |
| Look back on yesterday – graph | 2.6 (0.9)  n=13 | 3.3(1.1)  n=14 | 2.9 (1.1)  n=27 |
| Look back on yesterday – table | 3.2 (0.7)  n=13 | 4.1(0.6)  n=14 | 3.6 (0.8)  n=27 |
| Look back on yesterday – 4G scheme | 2.9 (1.0)  n=11 | 3.5 (0.9)  n=13 | 3.3 (1.0)  n=24 |
| Look back on yesterday – personalisation of the questions on the basis of Durall et al. (2017) | 2.5 (0.8)  n=13 | 2.8(0.9)  n=14 | 2.6 (0.8)  n=27 |
| Top 3 energy sources and leaks | 3.3 (0.9)  n=9 | 4.0(0.7)  n=14 | 3.7 (0.8)  n=27 |
| Reminders EnergyBalance | 2.5 (1.1)  n=13 | 3.4(0.9)  n=14 | 2.9 (1.1)  n=27 |
| Reminders Look back on yesterday | 2.6 (1.2)  n=13 | 3.4 (0.7)  n=14 | 3.0 (1.0)  n=27 |
| Elements phase 2 (scale 1-5): did the element helped you to determine appropriate strategies for the energy sources and leaks? |  |  | Number of participants filling in the questions in this phase  n=23 |
| BringBalance techniques | 2.6 (1.0)  n=9 | 4.1 (0.6)  n=14 | 3.5 (1.1)  n=23 |
| **How clear were the animation clips to you?** | 3.8 (1.3)  n=4 | 4.4 (0.6)  n=14 | 4.3 (0.8)  n=18 |
| **How clear did you find the text in the modules with the BringBalance techniques?** | 3.8 (0.5)  n=4 | 3.8 (0.6)  n=14 | 3.8 (0.5)  n=18 |
| **To which extent did the animation clips appeal to you?** | 3.8 (0.5)  n=4 | 4.1 (0.7)  n=14 | 4.0 (0.7)  n=18 |
| **How easy did you find it to learn the BringBalance techniques?** | 2.5 (1.0)  n=4 | 3.1 (0.9)  n=14 | 2.9 (0.9)  n=18 |
| **What did you think of the variation in the BringBalance techniques? Too little variance, just enough variance, too much variance.** | Too little = %  Just enough = 100%  Too much = %  n=3 | Too little = 21%  Just enough = 79%  Too much = %  n=14 | Too little = 18%  Just enough = 82%  Too much = %  n=17 |
| BringBalance techniques training days | 2.5 (0.7)  n=2 | 3.5 (0.8)  n=12 | 3.4 (0.8)  n=14 |
| BringBalance evaluation of training days | 2.5 (0.7)  n=2 | 3.4 (0.7)  n=11 | 3.2 (0.7)  n=13 |
| Techniques in your daily life | - | 3.6 (1.1)  n=9 | 3.6 (1.1)  n=9 |
| Biofeedback via the Inner Balance trainer during learning the techniques | 2.6 (1.3)  n=5 | 3.0 (1.0)  n=14 | 2.9 (1.0)  n=19 |
| **Did you find it useful to use the Inner Balance app while learning the BringBalance techniques?** | Yes = 67%  No = 33%  n=3 | Yes = 64%  No = 36%  n=14 | Yes = 65%  No = 35%  n=17 |
| HRV-measurements | 2.7 (0.6)  n=3 | 2.8 (1.1)  n=11 | 2.8 (1.0)  n=14 |
| Determine strategies for leaks - options | 2.7 (1.5)  n=3 | 3.2(0.8)  n=14 | 3.1 (0.9)  n=17 |
| Determine strategies for leaks – already have an idea | 3.0 (1.7)  n=3 | 3.0 (1.1)  n=8 | 3.0 (1.2)  n=11 |
| Determine strategies for leaks – strategy-database | 3.0 (1.0)  n=3 | 3.8 (0.7)  n=8 | 3.6 (0.8)  n=11 |
| Determine strategies for leaks – help from eCoach | - | 4.7 (0.6)  n=3 | 4.7 (0.6)  n=3 |
| Determine strategies for leaks – advice for a strategy | - | - | - |
| Determine strategies for sources | 3.0 (1.0)  n=3 | 2.9(0.7)  n=14 | 2.9 (0.7)  n=17 |
| More Zzleep? | 2.0  n=1 | 3.7 (1.0)  n=9 | 3.5 (1.1)  n=10 |
| Set implementation intentions (did this element helped you to experiment and evaluate whether the chosen strategy was the right one for the leak or source?) | 3.3 (1.5)  n=3 | 3.0(0.8)  n=14 | 3.1 (0.9)  n=17 |
| Phase 3 (scale 1-5): Did the element helped you in evaluating whether the chosen strategy was appropriate for the leak or source? |  |  | Number of participants filling in the questions in this phase  n=17 |
| Reminders with implementation intentions (did this element helped you to experiment and evaluate whether the chosen strategy was the right one for the leak or source?) | 1.0  n=1 | 2.6 (0.7)  n=11 | 2.4 (0.8)  n=12 |
| Experiment with the strategies | 2.0 (1.4)  n=2 | 2.9(1.0)  n=14 | 2.8 (1.0)  n=16 |
| Experiment with the BringBalance techniques | - | 2.5 (0.8)  n=11 | 2.5 (0.8)  n=11 |
| Experiment with the BringBalance techniques using the Inner Balance trainer | 1.0  N=1 | 2.8 (1.2)  n=11 | 2.7 (1.2)  n=12 |
| Experiment with sources to fill up the energy leaks | 4.0  N=1 | 2.9 (0.6)  n=13 | 3.0 (0.7)  n=14 |
| Experiment with an own thought off strategy | - | 3.2 (0.4)  n=5 | 3.2 (0.4)  n=5 |
| Experiment with strategies for energy sources | - | 3.1 (0.7)  n=12 | 3.1 (0.7)  n=12 |
| Strategy evaluation forms | - | 2.7 (0.8)  n=11 | 2.7 (0.8)  n=11 |
| Phase 3 EnergyBalance | - | 3.1 (0.8)  n=9 | 3.1 (0.8)  n=9 |
| Additional questions in EnergyBalance | - | 2.6 (0.7)  n=8 | 2.6 (0.7)  n=8 |
| Phase 4 (scale 1-5): Did the element helped you in evaluating whether the chosen strategy was appropriate for the leak or source? |  |  | Number of participants filling in the questions in this phase  n=13 |
| Evaluating the strategy – graph | - | 3.1 (1.2)  n=9 | 3.1 (1.2)  n=9 |
| Evaluating the strategy – table | - | 3.0 (0.9)  n=9 | 3.0 (0.9)  n=9 |
| Evaluating the strategy – questions | - | 2.8 (1.0)  n=12 | 2.8 (1.0)  n=12 |
| Evaluating if energy balance improved? – graph | - | 2.7 (0.9)  n=10 | 2.7 (0.9)  n=10 |
| Evaluating if energy balance improved? – table | - | 3.1 (1.2)  n=9 | 3.1 (1.2)  n=9 |
| Final advice | - | 3.3 (0.8)  n=12 | 3.3 (0.8)  n=12 |
