# Supplementary material for: How Reflective Automated e-Coaching Can Help Employees Improve Their Capacity for Resilience: Mixed Methods Study
Source: JMIR Hum Factors. 2023 Mar 10;10:e34331. doi: 10.2196/34331 (PMC10039404; doi:10.2196/34331)
Supplement: Multimedia Appendix 5 [file humanfactors_v10i1e34331_app5.docx]

## Multimedia Appendix 5 – Summary table of stimulators and stagnating factors for reflection per phase of reflection.

**Table.**

| Element | Stimulators | N* | Stagnating factors | N* |
| --- | --- | --- | --- | --- |
| *Phase 1 - Identification* | | | | |
| *EnergyBalance* | Perceived as an important element during reflection | - | Problems with recognizing energy sources and leaks | 5 |
|  | Energy sources and leaks could be filled in via the instructions in the EnergyBalance questionnaire | 10 | Difficulties with recognizing energy sources and leaks due to the term “Energy” | 2 |
|  | Writing down helps to gain insights | 4 | Blockade when writing down leaks and sources | 1 |
|  | Per part of the day leads to identification of ‘smaller’ leaks and sources | 3 | Boring due to the repetition of filling in the same questions | 3 |
|  | The focus on energy sources was experienced as positive | 4 | Writing down in own words includes a margin of error as you only report what you can recall | 1 |
|  | Most participants linked low energy levels to events related to a negative feeling and high energy levels to events related to a positive feeling | - |  |  |
|  | Repetition in filling in the same leaks and sources helps in observing a trend | 3 |  |  |
| *Look back on yesterday – 4G scheme* | Helps to understand personal indicators of leaks and sources which leads to better recognition later on | 2 | Problems recognizing physical and emotional indicators of leaks and sources | 4 |
|  | Questions stimulated to perform a more in depth reflection | 5 | Not recognizing physical and emotional indicators as he/she is more mentally present | 2 |
|  | Reflection via the 4G scheme a day later leads to being able to zoom out and notify more relevant aspects | 4 | Difficulties answering the questions due to difficulties recognizing indicators | 4 |
|  |  |  | Superfluous in relation to what has been filled in via the EnergyBalance | 5 |
| *Look back on yesterday – Table with overview of the data* | Important element that helped in gaining understanding | - |  |  |
|  | Contextual information from table led to a better reconstruction of the situation from the previous day | 4 |  |  |
|  | Look back on the day before is useful | 4 |  |  |
| *Look back on yesterday – graph with overview of the data* |  |  | One of the least important elements in the gain of understanding | - |
|  |  |  | Too little variance in the data visualized did not lead to a recall of the situation | 4 |
| *Top 3 energy sources and leaks* | The most important elements that helped in gaining understanding | - |  |  |
|  | The list regularly led to the observation of a trend: the most common leaks and sources were often the most important ones | 5 |  |  |
| *In general* | Elements in the app stimulates to think about the leaks and sources | 7 | Discussing the self-tracking data in a dialogue could have led to a higher level of reflection | 9 |
|  | Elements in phase 1 could be performed independently | 7 | The identified sources and leaks were sometimes too specific (due to EnergyBalance per part of the day but also due to 4G scheme) | 4 |
|  |  |  | Doubts about their thinking process | 3 |
| *Phase 2 – Strategy generation* | | | | |
| *The BringBalance*  *Techniques* | Important element in the gain of understanding of strategies | - | Need for confirmation from experts that techniques work | 3 |
|  | Principles of the techniques could be learned via the short clips | 12 |  |  |
|  | Examples in the clips were helpful to relate what and when to use in their situation | 3 |  |  |
|  | The deciding upon strategies initiated for some during watching the clips with the techniques | 2 |  |  |
| *BringBalance techniques training days* | Practicing was perceived as a crucial part to understand when and what strategy to use | 9 | Multitasking by focussing on breathing and mentally imaging was experienced as difficult to master by some | 3 |
|  | The ones that did use the reminders to train and evaluated the days of training in the app experienced it as helpful in the understanding when and what strategy to use | - | Reminders to train and the evaluation of the training days was not used very often due to (1) the aspect of time and (2) difficulties practicing without the presence of a relevant situation | (1)=5  (2)=2 |
|  |  |  | Period too short to master the techniques | 6 |
|  |  |  | Doubts if they performed the techniques in the right way | 4 |
| *Biofeedback via the Inner Balance trainer during learning the techniques* | The biofeedback was of added value during practicing. It guided them while practicing the techniques. | - | Difficulties in interpreting the results | 4 |
|  | The relationship between breathing-exercises and the effect on heart rate was made clear due to the biofeedback | 6 | Uncertainty about when to perform the measurement | 2 |
|  | Visualization of the biofeedback convinced them about the effectiveness of the technique | 8 | No improvement possible as measurements were good from the start | 3 |
| *Determine strategies* | Connecting strategies to the most important leaks and sources stimulates the mental process of how to integrate the techniques in daily life | 5 | More difficult to decide upon strategies for sources as techniques are perceived as having a better fit with the leaks | 4 |
|  | Strategies for energy sources were seen as just planning in more moments to perform the activity related to the source | 5 | Not experienced as having the freedom to also use the technique as maintenance of their EnerygBalance as it should be linked to a leaks or source | 4 |
| *Determine strategies for leaks – help from eCoach* | Was experienced as very helpful during the deciding of strategies | 3 | Not used very often by participants | - |
|  | It helps to structure your thoughts towards the right direction for a strategy | 3 |  |  |
|  | Most needed to use this tool twice during the deciding of the strategies | - |  |  |
| *Determine strategies for leaks – strategy-database* | Most participants chose the strategy-database as a tool to help them decide | - |  |  |
|  | Was perceived as helpful in deciding upon a strategy | 2 |  |  |
|  | Most needed this tool only ones during the deciding of strategies | - |  |  |
|  | The database was used as a refresher of what the techniques were about | 3 |  |  |
| *Implementation intentions* | Stimulates the mental process when to actually use the strategies in daily life | 2 | Steered to much towards setting up very specific implementation intentions | 4 |
|  | Stimulates the actual intention to use the strategies in daily life | 5 |  |  |
| *Setting up reminders with implementation intentions* |  |  | Hard to decide upon moments when it is useful to receive the reminders | 4 |
| *In general* | Understanding why the leaks or source effects the energybalance is important in order to be able to know what to do about your situation | 4 | Low-quality input from previous elements complicates the step of deciding upon strategies due to the fact that identified sources and leaks from phase 1 appeared to be irrelevant in phase 2 | 3 |
|  | Most were able to choose the strategies using the tools in BringBalance | 9 | Low-quality input from previous elements complicates the step of deciding upon strategies due to not mastering the techniques | 3 |
|  | The deciding upon strategies was already initiated for some during the identification of leaks and sources | 5 | Doubts existed if they had chosen the right strategies for their leaks and sources | 2 |
|  | Being attentive to indicators of sources and leaks, identified with the 4G scheme, was mentioned by a few as a prerequisite to understand when to apply the technique in daily life. | 2 | Discussing the results with someone as a check or receive advise what other options they should consider | 6 |
| *Phase 3 – Experimentation* | | | | |
| *Experimenting with strategies* | More easy to experiment with strategies for sources than leaks due to more structure in when to perform the strategies | 2 | Little experimentation took place due to: (1) leaks and sources did not occur anymore (2) time period was too short, and (3) too many techniques to experiment with. | (1)=6  (2)=4  (3)=4 |
| *Reminders with implementation intentions* | Reminders were triggers to start experimenting | 2 | Reminders for leaks and sources that occur randomly over time did not arrive at the right moment to trigger an action | 4 |
| *EnergyBalance phase 3* |  |  | Not often filled in by participants | - |
| *Strategy-evaluation form* | Perceived as a trigger to start the evaluation process of reflection | 3 | Too repetitive and generic questioning | 4 |
|  |  |  | Not necessary to fill in the forms each time depending on the specific strategy and/or the situation | 5 |
|  |  |  | Some participants asked themselves automatically the questions for evaluation after performing a strategy. Not necessary to report. | 2 |
| *In general* | The set-up in phase 3 made sense | 3 | The elements in this phase scored lowest in the process of reflection | - |
|  |  |  | Most started phase 3 later than planned | - |
|  |  |  | Wish to discuss their experiences during the experimentation with someone | 2 |
| *Phase 4 – Evaluation* | | | | |
| *Evaluation of the EnergyBalance and strategies* |  |  | Too little data presented in the overview to perform a meaningful evaluation | 6 |
|  |  |  | When they had known that they received such overviews, they would have collected more data in phase 3 | 2 |
|  |  |  | Wish to evaluate the strategies and energybalance at the end with someone | 3 |
| *Final advice* | This element received the highest score for the evaluation process | - |  |  |
| *In general* | Set-up of the elements for evaluation is useful to winded-up the programme | 7 | Doubts if the wind-up of the BringBalance program would lead to continuation of using the strategies in the future | 3 |
|  | Action was made for the continuation of integrating the strategies in daily life | 4 |  |  |
